# CXCL12/CXCR4 promotes inflammation-driven colorectal cancer progression through activation of RhoA signaling by sponging miR-133a-3p

**DOI:** 10.1186/s13046-018-1014-x

**Published:** 2019-01-24

**Authors:** Xinfeng Yu, Dong Wang, Xiaohui Wang, Shiyue Sun, Yuhang Zhang, Shuqing Wang, Rongrong Miao, Xiaoxue Xu, Xianjun Qu

**Affiliations:** 1 Department of Pharmacology, School of Basic Medical Sciences, Capital Medical University, Beijing, China; 2 Department of General Surgery, Xuan Wu Hospital, Capital Medical University, Beijing, China; 3 Department of Central Laboratory, Capital Medical University, Beijing, China

**Keywords:** CXCR4, RhoA, miR-133a-3p, lncRNA XIST, CAC

## Abstract

**Background:**

Activation of CXCL12/CXCR4 axis has been found to be associated with invasion and metastasis in many cancers. However, the underlying mechanism remains elusive. Increasing data highlight that non-coding RNAs are linked to CRC progression.

**Methods:**

The effects of CXCR4 were investigated using villin-CXCR4 transgenic mice model by flow cytometry assay, immunohistochemistry, and Western blot. The mechanism was explored through bioinformatics, luciferase reporter assay and RNA immunoprecipitation assay.

**Results:**

We found that high CXCR4 expression exacerbated colitis-associated cancer in villin-CXCR4 transgenic mice. *CXCR4*^*+/−*^*Apc*^*min/+*^ compound mutant mice demonstrated higher colorectal tumorigenesis than *Apc*^*min/+*^ mice. Furthermore, overexpression of CXCR4 was found to promote the epithelial-mesenchymal transition (EMT) and infiltration of myeloid-derived suppressor cells (MDSCs) and macrophages in colonic tissue, accelerating colitis-associated and *Apc* mutation-driven colorectal tumorigenesis and progression. Notably, miR-133a-3p was found to be significantly decreased in HCT116 cells overexpressing CXCR4 by miRNA sequencing. miR-133a-3p was proved to target RhoA, which is involved in cytoskeletal reorganization that drive cell motility. Importantly, CXCL12/CXCR4-induced upregulation of lncRNA XIST functioned as a ceRNA to sponge miR-133a-3p, thereby liberating the repression of RhoA by miR-133a-3p. The negative correlation of miR-133a-3p with RhoA was also confirmed in human CRC tissues and *CXCR4*^*+/−*^ mice.

**Conclusions:**

Our findings revealed the critical role of CXCR4 in promoting progression of inflammatory colorectal cancer through recruiting immunocytes and enhancing cytoskeletal remodeling by lncRNA XIST/ miR-133a-3p/ RhoA signaling. These results provide novel potential therapeutic targets for hindering CXCL12/CXCR4-induced CRC progression.

**Electronic supplementary material:**

The online version of this article (10.1186/s13046-018-1014-x) contains supplementary material, which is available to authorized users.

## Background

Colorectal cancer (CRC) is the third most common cancer worldwide. The mortality of CRC is principally attributable to the development of liver metastasis that is a critical determinant of poor prognosis of CRC patients [1]. CRC involves multi-factorial and multistep progression in which abnormal expression of Chemokine (C-X-C motif) receptor 4 (CXCR4) has been demonstrated to play a crucial role in the invasion and liver metastasis of CRC [2]. CXCR4 belongs to G protein-coupled receptor superfamily, which selectively binds to Stromal cell-derived factor 1 (SDF-1), also known as CXCL12 to promote cancer metastasis [3]. However, the underlying mechanism has not been fully understood. Some signaling pathways such as Wnt/β-catenin and PI3K-AKT are reported to contribute to the CXCR4-mediated invasion and angiogenesis in cancers [4, 5]. Recent data highlight that microRNAs (miRNAs) are closely associated with CRC progression [6]. MiRNAs belong to a cluster of highly conserved 22-nucleotide single-stranded RNAs that trigger mRNA degradation or translational inhibition by forming the RNA-induced silencing complex (RISC) [7]. However, whether miRNAs are involved in CXCR4-driven invasion and metastasis of CRC remains poorly explored.

It is recently proposed that a large number of non-coding RNAs might function as molecular sponges for miRNAs and hence functionally liberate other RNA transcripts targeted by active miRNAs [8]. Long non-coding RNAs (lncRNAs), longer than 200 nucleotides, control gene expression by diverse modes, including miRNA sponging, epigenetic modification and mRNA stabilization [9]. In CRC, aberrant expression of lncRNAs promotes tumorigenesis and metastasis by acting as competitive endogenous RNAs (ceRNAs) and “miRNA sponges” to antagonize their functions and leads to the de-repression of their endogenous targets [10, 11]. Recently lncRNA X-inactive-specific transcript (XIST) has attracted more attention and it was demonstrated that lncRNA XIST was significantly increased in CRC and could be a potential oncogene in CRC [12]. LncRNA XIST is transcribed from the X inactivation center of the X chromosome that directly binds to polycomb repressive complex 2 (PRC2), which is the epigenetic complex responsible for the trimethylation of histone H3 at Lys27 (H3K27me3), silencing the whole chromosome [13]. LncRNA XIST was found to expedite metastasis by competing for miR-200b-3p to modulate the expression of ZEB1 [14]. It could also sponge miR-34a to promote colon cancer progression via Wnt/β-catenin signaling pathway [15].

Increasing data indicated that Rho signaling contributes to tumor invasion and metastasis by actin cytoskeletal reorganization. RhoA is capable of mediating stress fiber formation and generating contractile force needed for retraction of the trailing edge during migration. ROCK is a major mediator of RhoA function. Inhibition of ROCK blocks the formation of RhoA-mediated actin cytoskeletal structures. Many studies have shown that RhoA/ROCK-mediated cytoskeletal regulation plays a key role in cancer metastasis. Therefore, targeting RhoA/ROCK signaling-associated miRNAs could be useful strategy for inhibiting migration of cancer cells in treatment of cancer metastasis.

In this study, using villin-CXCR4 transgenic mice, we found that overexpressing CXCR4 enhanced tumor load in azoxymethane/dextran sulfate sodium (AOM/DSS)-treated mice and *Apc*^*min/+*^ mice. CXCR4 promotes the progression of colitis-associated cancer (CAC) by enhancing EMT and recruiting myeloid-derived suppressor cells (MDSCs) and macrophages. In vitro assay using cell model by overexpressing and silencing of CXCR4, we unraveled that miR-133a-3p was significantly reduced in colorectal cancer cells overexpressing CXCR4. We demonstrated that RhoA, a GTPase that facilitates actin polymerization, was the direct target gene of miR-133a-3p. Furthermore, lncRNA XIST functions as a ceRNA sponging miR-133a-3p, thereby de-repressing target gene of RhoA. Our results shed new lights on the progression of CRC driven by inflammation and cytoskeletal reorganization through the lncRNA XIST/ miR-133a-3p/ RhoA signaling pathway.

## Materials and methods

### Construction of mice models

Villin-CXCR4 transgenic mice (CXCR4^+/−^ Tg) were generated by Cyagen Biosciences Inc. (Guangzhou, China) overexpressing CXCR4 in intestinal epithelial cells (IEC) under the control of villin promoter.

C57BL/6 J male mice heterozygous for *Apc* allele (*Apc*^*Min/+*^) were purchased from Jackson Laboratory (Shanghai, China). *CXCR4*^*+/−*^*Apc*^*Min/+*^ mice were established by mating *Apc*^*Min/+*^ males with CXCR4^+/−^ females. Genotyping was performed by allele specific PCR assays using the primers described in Additional file 1: Table S1. Mice were maintained in the Animal Care Facility of Capital Medical University. Housing and care of the animals were in accordance with the NIH Guidelines for the Care and Use of Laboratory Animals. All experiments related to animals were approved by Animal Welfare Committee of Capital Medical University.

### AOM/DSS-induced colonic tumorigenesis model

The establishment of AOM/DSS mouse model was conducted as previously described [16]. In brief, 8 weeks old C57BL/6 mice (*n* = 8 per group) were treated by a single intraperitoneal injection of AOM (10 mg/kg, Sigma-Aldrich) and subsequent oral administration of 1% DSS (MP Biomedicals) in drinking water ad libitum for 7 consecutive days and then removed for 14 days intervals. Three cycles of DSS are performed to establish this model. To inhibit CXCR4, AMD3100 (2.5 mg/kg, Sigma-Aldrich) was administered intraperitoneally daily for nine consecutive weeks. Mice were monitored twice a week for clinical signs of illness including rectal bleeding, rectal prolapse and body weight loss. *CXCR4*^*+/−*^*Apc*^*Min/+*^ mice (aged at 12 weeks, *n* = 15) were treated with 3 cycles of 1% DSS and sacrificed at age of 22, 26 and 30 weeks respectively. DSS-induced colitis was scored as disease activity index (DAI) obtained based on weight loss, stool consistency, and bloody excreta as follows: weight loss score = 0: < 1%, 1: 1–5%, 2: 5–10%, 3:10–15%, 4: > 15%; stool consistency score = 0: normal, 2: loose, 4: diarrhea; blood in excreta score = 0: normal; 2: reddish, 4: bloody [17]. Upon sacrifice, colons and intestines were excised and opened longitudinally to count tumor numbers. Size of tumors was measured with a caliper to evaluate intestinal tumor development. Colons were further processed for histopathological analysis.

### Immunohistochemistry and immunofluorescence staining

Colorectal tissues and tumors were fixed in 10% formalin followed by paraffin embedding. Tissue sections of 5 μm were stained with hematoxylin & eosin (HE) for pathological evaluation using standard procedures. Immunohistochemistry (IHC) was performed using the antibodies of E-cadherin (3195), Vimentin (5741), active β-catenin (8814) (CST), CXCR4 (ab1670) (Abcam), RhoA (sc-418) (Santa Cruz), F4/80 (GB11207), CD4 (GB13064), CD8 (GB11068), CD68 (GB11067) (Servicebio) according to manufacturer’s instructions. The results of the IHC analysis were taken with a digital slide scanning system (Pannoramic Scan, 3DHISTECH Ltd.). The results were semi-quantified of mean density (IOD/area) by image-pro plus 6 software (IPP, USA).

For immunofluorescence staining of cytoskeleton, Rhodamine-labeled phalloidin and nuclear labeling with DAPI were performed as instructed by cytoskeleton Inc. briefly, SW620 cells overexpressing CXCR4 and vector control (GFP-labeled) were fixed in 4% paraformaldehyde, blocked with 2% bovine serum albumin, and incubated with Rhodamine-labeled phalloidin at 4 °C overnight. Nuclei were labeled with DAPI. Fluorescence images were captured using a Leica confocal scanning system.

### Immunoblotting

Colon tissue fractions or cell pellets were lysed in RIPA buffer (Beyotime, Nanjing, China) supplemented with 1 mM PMSF and complete protease inhibitor mixture (Roche Pharmaceuticals) on ice. Total protein was quantified by using Pierce BCA Protein Assay Kit (Thermo Scientific, Germany). Equal amounts of protein lysates were resolved by SDS-PAGE and transferred to PVDF membranes (Millipore, Merck). The membranes were immunoblotted at 4 °C with primary antibodies against E-cadherin (3195), Vimentin (5741), Snail (3879), c-Myc (5605), MMP7 (3801), β-catenin (8480), active β-catenin (8814), IL-1 (12242), IL-6 (12912), COX-2 (12282), P-STAT3 (9145), STAT3 (12640), P-JAK2 (3776), JAK2 (3230), RhoA (2117), P-FAK (3284), FAK (13009), ROCK1 (4035), P-MLC (3671), MLC (8505), (CST) and CXCR4 (ab1670) (Abcam) followed by HRP-conjugated secondary antibodies. Immunoreactive products were visualized using Fluorchem FC3 system (Proteinsimple, USA) by chemiluminescence (Millipore, USA) and quantified by densitometry using Alphaview software. Densitometric analyses of bands were normalized with β -actin functioning as a loading control.

### RNA extraction and RT-qPCR

Total RNA was extracted using TRIzol reagent (Invitrogen). Reverse transcription of total miRNA was performed by using a miScript reverse transcription kit (Qiagen) in accordance with manufacturer’s protocol. MiScript SYBR Green PCR kit (Qiagen) and miR-133a specific primers were used to determine the expression of mature miRNAs. RNU6B was used as an internal control.

To determine mRNA expression, reverse transcription was carried out using SuperScript III First-Strand Synthesis System (Invitrogen) according to manufacturer’s instruction. Real-time PCR was performed in triplicate using Powerup SYBR Green qPCR master mix in an ABI 7500 fast real-time PCR system (Applied Biosystems). The thermal cycling was initiated by polymerase activation step for 10 min at 95 °C followed by 40 cycles of denaturation (95 °C for 15 s) and annealing/extension (60 °C for 1 min). Target gene expression levels were normalized to GAPDH and determined by a previously described method [18].

### Flow cytometry analysis

The immunocytes isolated from blood and inflamed colonic tumors were subjected to flow cytometry analysis according to previously described method [19]. For multicolor flow cytometry analysis, cells were stained with indicated antibodies (Additional file 1: Table S2) and acquired on a BD LSR Fortessa flow cytometer. Briefly, the immnocytes were incubated for 30 min on ice with the appropriate combination of antibodies in staining buffer (Biolegend) and the data were analyzed using TreeStar Flowjo software.

### Enzyme-linked immunosorbent (ELISA) assay

The level of IL-1, IL-6 and tumor necrosis factor alpha (TNF-α) was quantified by using ELISA kits (Immunoway, USA) according to manufacturer’s protocols.

### Cell culture and cell transfection

Human colorectal adenocarcinoma cell lines SW620 and HCT116 were purchased from American Type Culture Collection (ATCC, Rockville, MD). Cells were grown in RPMI 1640 medium containing 10% fetal bovine serum (FBS) and penicillin/streptomycin at 37 °C in a humid atmosphere (5% CO2). Cancer cells were infected with lentivirus expressing CXCR4 (LV-CXCR4) or transfected with CXCR4 siRNA as described previously [20]. To knockdown lncRNA XIST, 200 nM of XIST siRNA with oligonucleotide sequences 5′- GCUGCACUAAUUGACUAAUTT-3′ were used to transfect cells for 48 h (GenePharma, Shanghai, China). To determine the roles of miR-133a, cells were transfected with 100 nM of miR-133a mimics or inhibitors (GenePharma Shanghai, China) for 48 h by using lipofectamine 2000 (Invitrogen) according to manufacturer’s instructions.

### RNA sequencing

Total RNA was extracted from HCT116 cells infected with lentiviral CXCR4 (Genechem, Shanghai) and stimulated with CXCL12 using Trizol reagent (Invitrogen). RNA integrity and concentration was assessed by using the RNA Nano 6000 assay kit of the Bioanalyzer 2100 system (Agilent, CA). Library construction and sequencing were performed on an Illumina HiSeq2500 sequencer according to manufacturer’s specifications (Illumina) at Annoroad Gene Technology (Beijing, China). miRNAs with *P* < 0.05 and |log2_ratio| ≥ 1 are identified as differentially expressed miRNAs.

### Plasmid construction and luciferase reporter assay

To confirm that RhoA was the target gene of miR-133a, RhoA gene containing miR-133a binding sites was synthesized and digested by *Xba* I and cloned into GV272 vector (GeneChem, Shanghai). Similarly, XIST gene containing miR-133a binding sites was cloned into GV272 vector (GeneChem, Shanghai) to make the luciferase constructs. Wild type and mutant inserts were confirmed by sequencing. GV272 empty vector was used as control (Con081).

The luciferase reporter assay was performed as previously described [20]. Briefly, HCT116 cells were co-transfected with wild type or mutant RhoA-3’UTR-Luc/ LncRNA XIST-Luc firefly luciferase constructs (100 ng) and 40 nM miR-133a mimics or inhibitors using lipofectamine 2000 reagent. 2 ng of pRL-SV40 plasmid was transfected to monitor transfection efficiency. Luciferase activity was determined by a dual-luciferase reporter assay system (Promega).

### RNA immunoprecipitaion (RIP) assay

RIP assay was performed by the EZ-Magna RIP kit (Millipore) according to the instructions. Briefly, cells were lysed in complete RIP lysis buffer. Then, the cell lysate was incubated with RIP buffer containing magnetic beads conjugated to a human anti-Ago2 antibody (Millipore). The precipitates were incubated with proteinase K with shaking to digest proteins and the immunoprecipitated RNA was isolated. Subsequently, the purified RNA was subjected to RT-qPCR analysis.

### Human CRC tissue specimens

The experiment was approved by the Institutional Review Board of Xuan Wu Hospital of Capital Medical University. The informed consents were obtained from all patients. Human CRC tissue specimens and adjacent normal mucosa were obtained from Xuan Wu Hospital of Capital Medical University (Beijing, China) and were confirmed by pathological analysis. The clinical information was shown in Additional file 1: Table S3.

### Rho activation assay

RhoA GTP levels were assessed using a Rho-binding domain (RBD) affinity precipitation assay (Cytoskeleton, Denver, CO). Briefly, HCT116 and SW620 cells were grown to 80% confluence in 100 mm tissue culture plates and serum-starved overnight. Cells were infected with lentivirus expressing CXCR4 (LV-CXCR4) or transfected with CXCR4 siRNA or miR-133a-3p mimics before stimulation with 50 ng/ml CXCL12 for 12 h. Cells were solubilized in lysis buffer and cleared by centrifugation. Cleared lysates were incubated with RBD-GST beads for 1 h at 4 °C to bind RhoGTP. The beads were washed, eluted in SDS-sample buffer and analyzed by immunoblotting with anti-RhoA monoclonal antibody.

### Statistical analysis

All data were presented as mean ± SD and statistical data were analyzed using SPSS 12.0. Statistical differences among multiple groups were evaluated by one-way analysis of variance (ANOVA) followed by Dunnett (multiple comparisons to the same control) post hoc tests. Student’s *t* test was used to compare differences between two groups. The Spearman’s correlation was used to evaluate potential correlations between miR-133a and RhoA expression in paired CRC tissues. A *P* < 0.05 was considered to indicate statistical significance.

## Results

### Overexpression of CXCR4 exacerbated AOM/DSS-induced CAC in mice

**Fig. 1:**
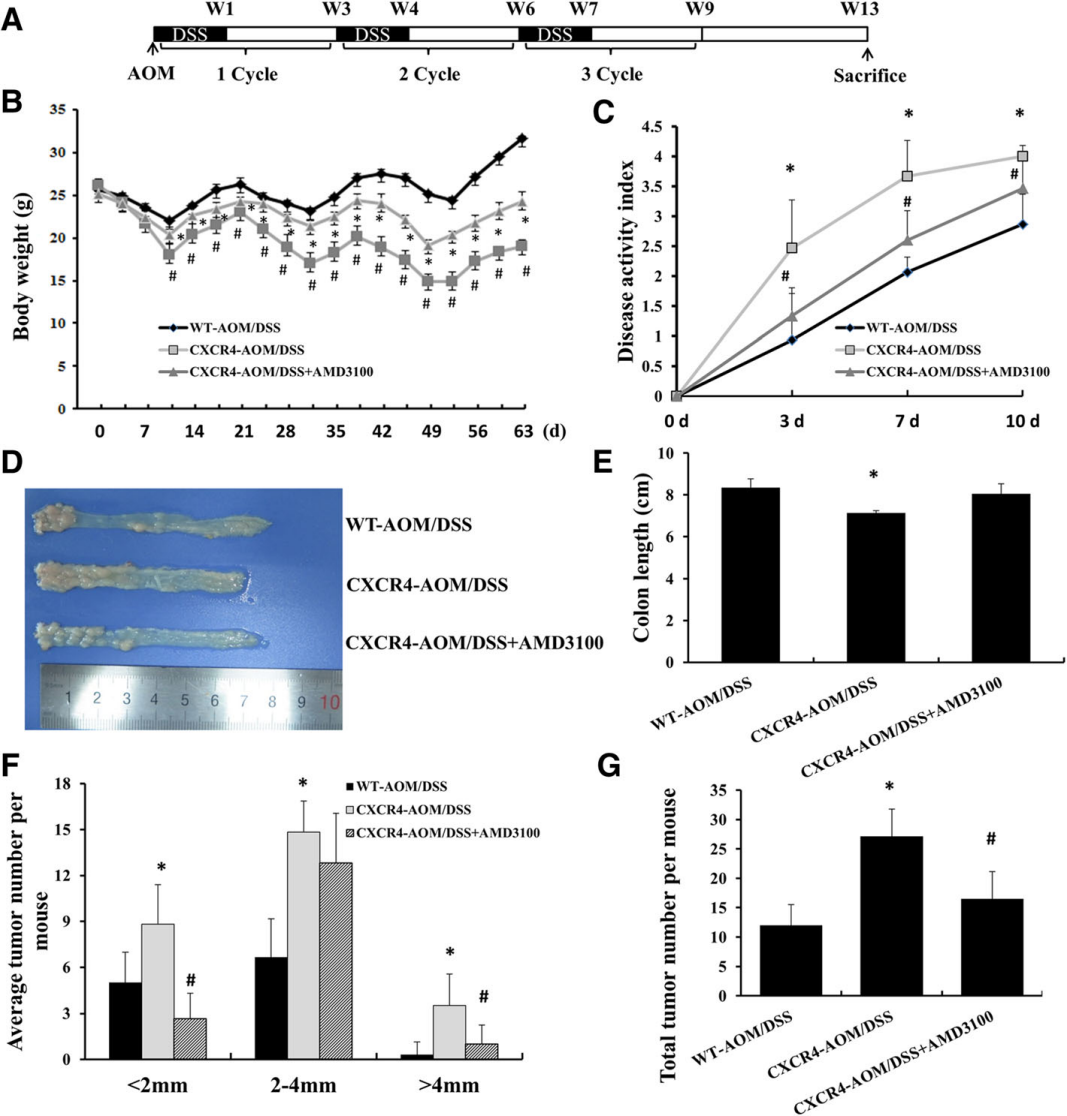
*CXCR4*^*+/−*^ transgenic mice displayed enhanced AOM/DSS-induced CAC. **a** The schematic regimen of colitis-associated cancer model by treatment with 3 cycles of AOM/DSS. **b** Wild type C57BL/6 J mice and *CXCR4*^*+/−*^ mice were treated with AOM/DSS with or without AMD3100 for 63 days. Body weight changes were measured twice a week. **c** Disease activity index was evaluated in the mice. **d** Representative images of colons from AOM/DSS-treated WT (*n* = 8), *CXCR4*^*+/−*^ (*n* = 8) mice and *CXCR4*^*+/−*^ mice treated with AMD3100 (*n* = 8). **e** Colon lengths were measured following treatment. **f** Average colon polyp counts per mouse were classified by polyp size. **g** Total polyp counts per mouse were examined in different groups (*n* = 8). *P < 0.05 vs. WT mice. ^#^*P* < 0.05 vs. *CXCR4*^*+/−*^ mice

To investigate the role of CXCR4 in colitis-associated cancer, wild type (WT) and intestine epithelial cell specific CXCR4 transgenic mice (CXCR4^+/−^) were treated with AOM and DSS as described in Methods (Fig. 1a). As shown in Fig. 1b, DSS-induced body weight loss in *CXCR4*^*+/−*^ mice was more obvious than in WT mice, whereas CXCR4 antagonist AMD3100 attenuated body weight loss during colitis. In *CXCR4*^*+/−*^ mice, AOM/DSS exposure induced bloody stool, diarrhea and body weight loss as indicated by higher disease activity index (DAI) scores than WT mice (Fig. 1c). Moreover, DSS induced severe inflammation in colon, resulting in shortening of colon length (Fig. 1d, e), which could be impaired by AMD3100. In addition, overexpression of CXCR4 dramatically increased chronic inflammation and tumor burden, the number and size of colonic adenocarcinoma in *CXCR4*^*+/−*^ mice were larger than that of WT mice, while AMD3100 significantly reduced the number of colonic adenocarcinomas that less than 2 mm or more than 4 mm in diameter (Fig. 1f, g). These results suggest that overexpression of CXCR4 exacerbates inflammation-driven tumorigenesis in colon.

### *CXCR4*^*+/−*^*Apc*^*min/+*^ compound mutant mice exhibited more tumor load than *Apc*^*min/+*^ mice

**Fig. 2:**
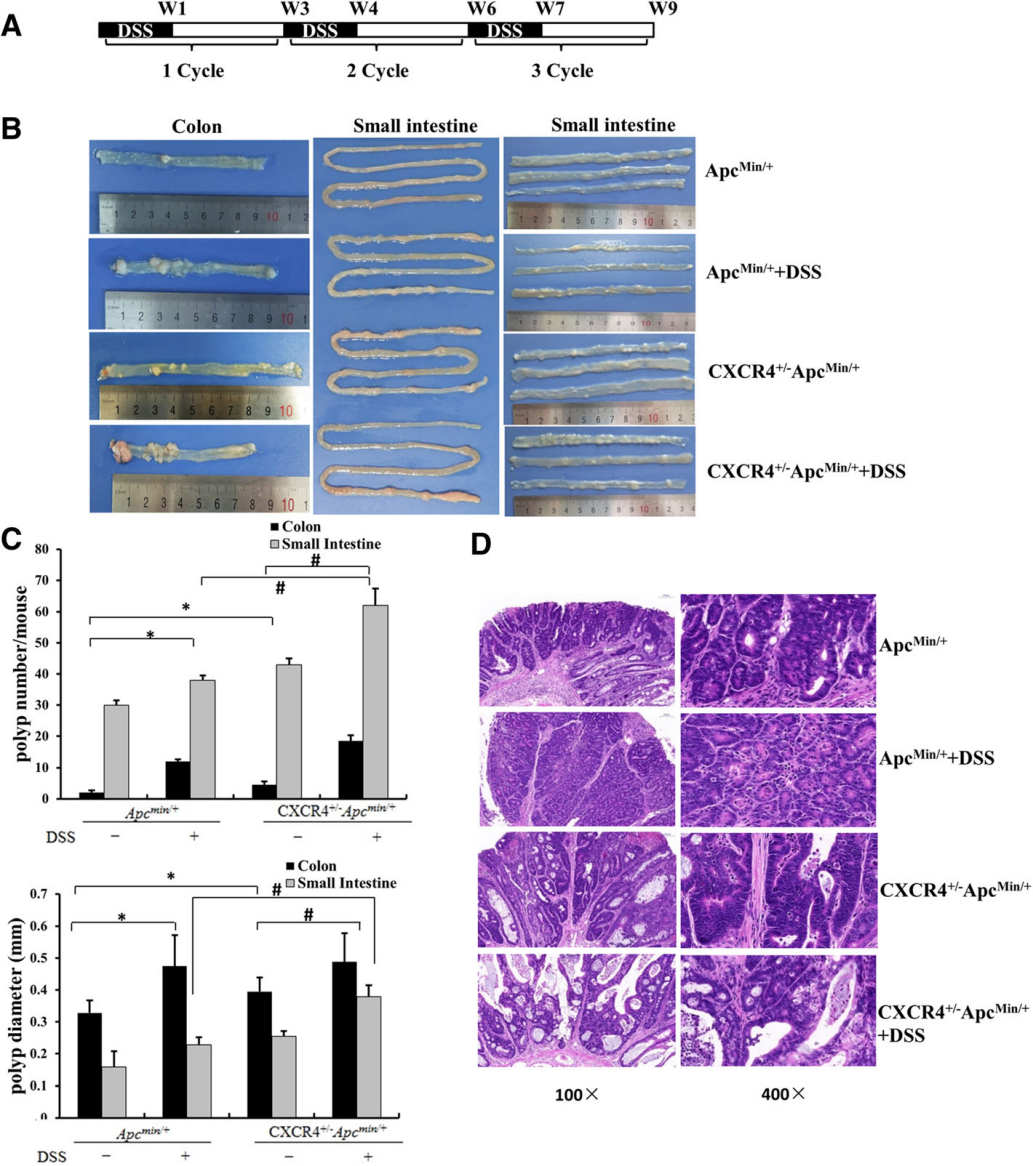
*CXCR4*^*+/−*^*Apc*^*min/+*^ compound mutant mice exhibited more tumor load than *Apc*^*min/+*^ mice. **a** The schematic regimen of inflammatory cancer in *Apc*^*min/+*^ and *CXCR4*^*+/−*^*Apc*^*min/+*^ mice by treatment with 3 cycles of 1% DSS. **b** *Apc*^*min/+*^ and *CXCR4*^*+/−*^*Apc*^*min/+*^ mice (aged at 12 weeks, *n* = 15) were treated with or without 3 cycles of 1% DSS. At age of 30 weeks, the mice were sacrificed and representative images of intestine polyps were shown. **c** Average number and size of polyps of colon and small intestine were determined and statistical analysis was performed (*n* = 5 for each group). *P < 0.05 vs. *Apc*^*min/+*^ mice. ^#^*P* < 0.05 vs. *Apc*^*min/+*^ and *CXCR4*^*+/−*^*Apc*^*min/ +*^mice respectively as indicated. **d** Representative H&E staining of colonic tumors (*n* = 5 for each group)

To explore the role of CXCR4 in inflammation-associated tumorigenesis in a spontaneous colon cancer model, *CXCR4*^*+/−*^ transgenic mice were bred onto *Apc*^*min/+*^ background to generate *CXCR4*^*+/−*^*Apc*^*min/+*^ compound mutant mice. *Apc*^*min/+*^ mice that have a germ-line mutation in *Apc* spontaneously develop a large number of tumors in small intestine, but very few polyps in colon [21]. Thus, *Apc*^*min/+*^ mouse has been utilized as a spontaneous colon cancer model to identify genetic elements triggering tumor initiation. The representative genotyping results were shown in Additional file 2: Figure S1. *Apc*^*min/+*^ and *CXCR4*^*+/−*^*Apc*^*min/+*^ mice (aged at 12 weeks) were treated with 3 cycles of 1% DSS as indicated in Fig. 2a. As expected, *Apc*^*min/+*^ mice developed average 30 polyps per small intestine and 2 adenomas per colon. Notably, *CXCR4*^*+/−*^*Apc*^*min/+*^ compound mutant mice develop on average 43 polyps and 5 adenomas per colon. Compared with *Apc*^*min/+*^ mice, where the location of adenomas is almost exclusively in small intestine, *CXCR4*^*+/−*^*Apc*^*min/+*^ mice exhibited more tumor load in colon at age of 30 weeks in addition to the increased adenoma in small intestine (Fig. 2b). The results implied that overexpression of CXCR4 exacerbated colitis-associated tumorigenesis in *Apc*^*min/+*^ mice. Moreover, the genetic effects of CXCR4 on tumorigenesis and progression were potentiated by DSS-induced inflammation. As shown in Fig. 2c, DSS increased adenoma number dramatically in the colon and modestly in the small intestine. It was also noted that colon and small intestine polyps developed in a time-dependent manner. *CXCR4*^*+/−*^*Apc*^*min/+*^ mice treated with 3 cycles of DSS developed dramatically more adenomas than their littermate *Apc*^*min/+*^ mice at age of 30 weeks. Similar effects were also seen at early ages (22 weeks and 26 weeks, Additional file 3: Figure S2).

**Fig. 3:**
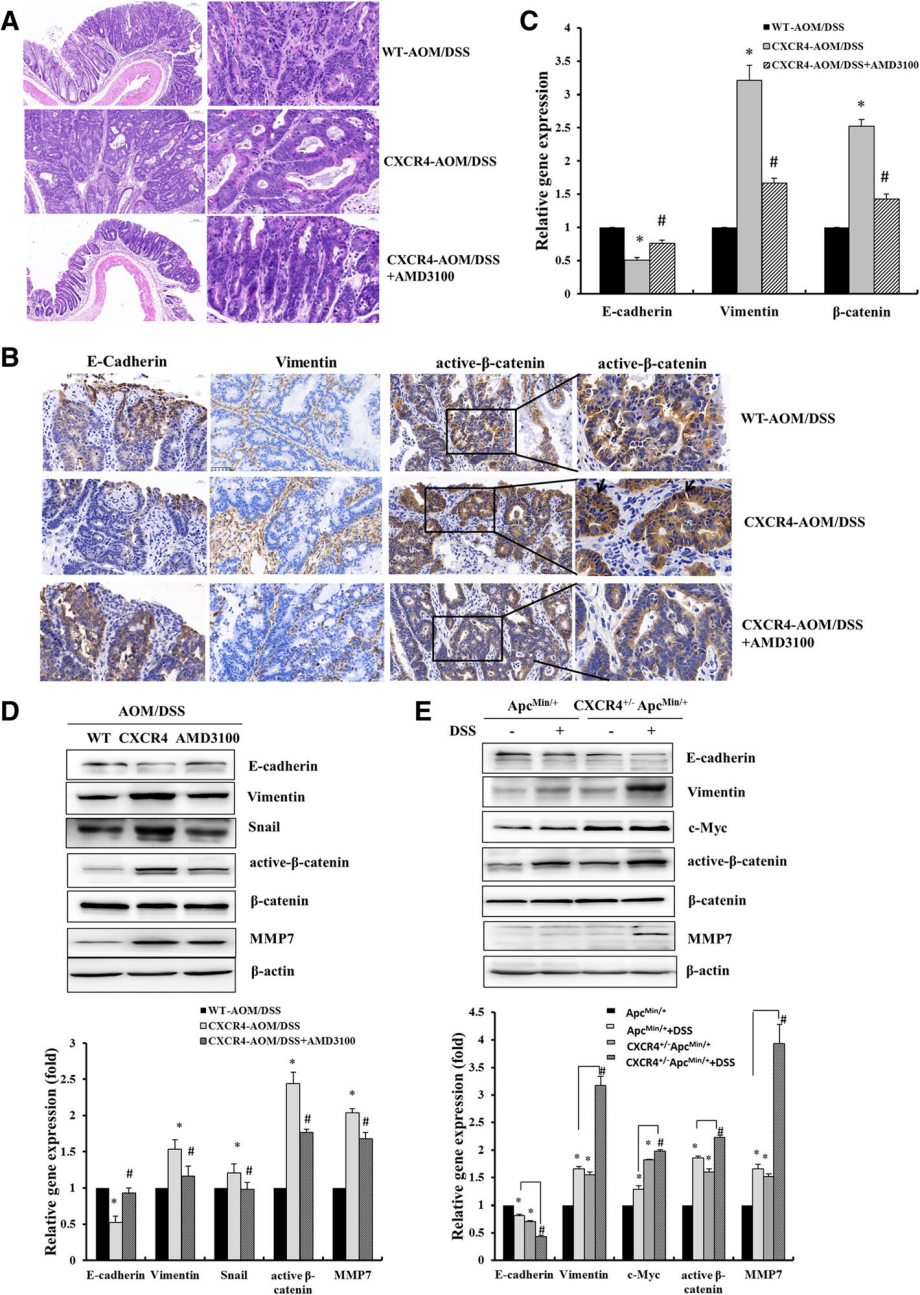
CXCR4 enhanced CRC progression by inducing EMT and Wnt-β-catenin activation. **a** Representative H&E staining of colonic tissue with tumors from AOM/DSS treated WT and *CXCR4*^*+/−*^ mice with or without AMD3100 treatment. **b** Representative images were shown to indicate the expression of E-cadherin, Vimentin, and active-β-catenin in different groups (*n* = 5) determined by immunohistochemistry assay (400 ×). Magnification of active β-catenin in black box was indicated and arrows pointed the nuclear staining of β-catenin. **c** Immunohistochemistry results were semi-quantified by image-pro plus 6 software and statistical analyses were performed (*n* = 5 for each group). *P < 0.05 vs. WT mice. ^#^*P* < 0.05 vs. *CXCR4*^*+/−*^ mice. **d**, **e** Levels of E-cadherin, vimentin, Snail, c-Myc, active-β-catenin/β-catenin, MMP7 normalized to β-actin were determined by Western blotting assay and statistical analyses were also performed

Histological analysis revealed that *CXCR4*^*+/−*^*Apc*^*min/+*^ mice exhibited larger adenomas with atypical dysplasia than *Apc*^*min/+*^ mice. DSS increased the infiltration of inflammatory cells into the lamina propria, loss of crypts and epithelial cell denudation (Fig. 2d). The polyps developed in AOM/DSS-treated group were identified adenocarcinomas. AOM/DSS-treated *CXCR4*^*+/−*^ mice exhibited much larger adenocarcinomas with disordered crypt structure and glandular lumens than WT mice carrying micro-adenomas with atypical dysplasia. AMD3100 significantly attenuated AOM/DSS-induced adenocarcinoma and restored to organized crypts and small adenoma in *CXCR4*^*+/−*^ mice (Fig. 3a).

### CXCR4 enhanced CRC progression by promoting EMT and Wnt-β-catenin activation

We next sought to determine whether high CXCR4 expression promotes tumor progression to an invasive phenotype in vivo*.* Immunohistochemistry was performed to detect the expression levels of key genes related to epithelial-to-mesenchymal transition (EMT) phenotype. EMT is a process by which epithelial cells lose their cell polarity and gain migratory and invasive phenotype. It is characterized by loss of epithelial markers E-cadherin and upregulation of mesenchymal marker vimentin. As expected, *CXCR4*^*+/−*^ mice revealed a pronounced downregulation of E-cadherin in the cell membrane of intestine epithelial cells and upregulation of cytoplasmic vimentin in the mesenchymal cells. These results suggest that CXCR4 enhanced the invasion and metastasis of CAC by promoting EMT phenotype, which could be impaired by AMD3100 treatment (Fig. 3b, c). Consistently, we confirmed the loss of E-cadherin and upregulation of vimentin expressiom in colon tissue of *CXCR4*^*+/−*^ mice by Western blot analysis (Fig. 3d, e). Furthermore, the marked activation of Wnt-β-catenin signaling pathway was also found in *CXCR4*^*+/−*^ mice compared to that of WT mice by immunohistochemistry and Western blot analysis, showing more nuclear staining of β-catenin and upregulation of active β-catenin in colonic tissue of *CXCR4*^*+/−*^ mice (Fig. 3b-e).

### CXCR4 enhanced CRC progression by increasing inflammatory cytokines and recruiting immune suppressive cells

**Fig. 4:**
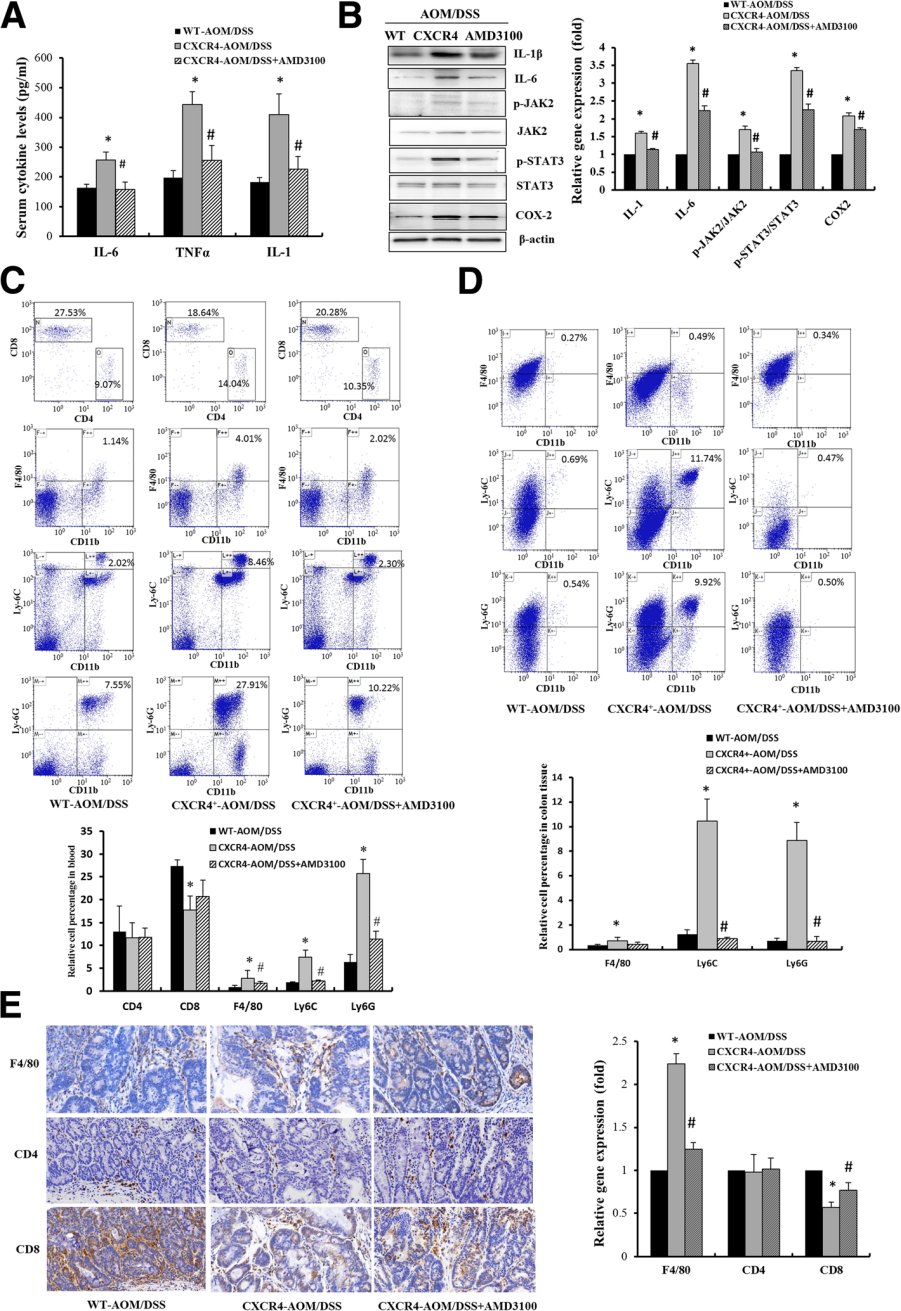
CXCR4 exacerbated CRC progression by increasing inflammatory cytokines and recruiting immune suppressive cells. **a** ELISA was performed to examine the levels of cytokine IL-1, IL-6 and TNFα (*n* = 5 for each group). **b** The levels of IL-1β, IL-6, p-JAK2/JAK2, p-STAT3/STAT3 and COX2 were determined by Western blot assay. Bar graphs indicated the relative levels of these proteins normalized to β-actin. *P < 0.05 vs. WT mice. ^#^*P* < 0.05 vs. *CXCR4*^*+/−*^ mice. **c**, **d** The percentages of gated CD4^+^, CD8^+^ T-cells, CD11b^+^F4/80^+^ macrophages, CD11b^+^Ly6C^+^, CD11b^+^Ly6G^+^ MDSCs immune cells in the blood and colonic tissues of AOM/DSS treated mice were subjected to flow cytometry analysis (*n* = 5 for each group). **e** Representative images were shown to indicate the expression of F4/80, CD4^+^, CD8^+^ T-cells in different groups (*n* = 5) determined by immunohistochemistry assay (400 ×)

CAC was characterized by elevation of pro-inflammatory cytokines and a massive infiltration of leukocytes in the intestinal mucosa. ELISA assay revealed a robust increase of serum IL-1, IL-6 and TNFα levels in *CXCR4*^*+/−*^ mice. Consistently, Western blot assay indicated that the expression of IL-1 and IL-6 was significantly increased and the well-established JAK2-STAT3 inflammatory signaling was strikingly activated in *CXCR4*^*+/−*^ mice compared to those of WT mice. The CXCR4-induced CRC progression was attenuated by AMD3100 (Fig. 4a, b).

MDSCs and tumor-associated macrophages (TAM) could enhance tumor progression by facilitating the growth and migration of tumor cells and suppression of immune response [22]. To determine whether CXCR4 promotes the recruitment of immune cells from the circulatory system into the inflamed intestinal tissue, we used cell surface markers to define the immunocyte profiles in blood and colonic tissues. Overexpression of CXCR4 dramatically increased the percentages of macrophage (CD11b^+^F4/80^+^), granulocytic MDSCs (G-MDSCs, CD11b^+^Ly6G^+^) and monocytic MDSCs (M-MDSCs, CD11b^+^Ly6C^+^) in the blood (Fig. 4c). Importantly, *CXCR4*^*+/−*^ mice exhibited more accumulation of macrophages and MDSCs in the intraepithelial and lamina propria of colonic tissues than WT mice. AMD3100 strongly abrogated the AOM/DSS-induced the infiltration of these immunocytes in colonic tissues (Fig. 4d). The massive infiltration of macrophages (F4/80^+^) in colonic tissues of AOM/DSS-induced *CXCR4*^*+/−*^ mice was also evaluated by IHC staining as presented in Fig. 4e. These results suggest that CXCR4 is responsible for the recruitment of macrophage and MDSCs from circulatory system to inflamed colonic mucosa and tumors. MDSCs have been shown to play an important role in immune evasion via suppressing the activation and proliferation of T cells and NK cells [23]. The recruited MDSCs trigger tumor progression primarily via suppressing CD8^+^ T-cell cytotoxicity against tumor cells. Since AOM/DSS-induced massive infiltration of MDSCs in colonic tissue, thus, it was conceivable that a lower percentage of CD8^+^ T-cells were observed in CAC of *CXCR4*^*+/−*^ mice compared with WT mice by flow cytrometry and IHC analysis (Fig. 4c, e).

Immune suppressive MDSCs and macrophages are key participants of inflammatory tumor microenvironment where they can promote tumor initiation and metastasis. To determine the effect of CXCR4 on inflammatory microenvironment in *Apc*^*min/+*^ mice, flow cytometry was employed to examine MDSCs and macrophages in blood and colonic tissues of *CXCR4*^*+/−*^*Apc*^*min/+*^ and *Apc*^*min/+*^ mice. As presented in Additional file 4: Figure S3A, robust increased numbers of M-MDSCs (CD11b^+^Ly6C^+^) and macrophages (CD11b^+^F4/80^+^) and diminished numbers of CD8^+^ T-cells were observed in the blood of CXCR4^+/−^*Apc*^*min/+*^ mice compared with *Apc*^*min/+*^ mice. Notably, the significant increase of MDSCs and macrophages in blood was potentiated by DSS. Importantly, there were greater infiltration of both MDSCs and macrophages in colonic mucosa of *CXCR4*^*+/−*^*Apc*^*min/+*^ than *Apc*^*min/+*^ mice, which was strengthened by DSS (Additional file 4: Figure S3B). Expectedly, the percentage of CD8^+^ T-cells were significantly reduced in CXCR4^+/−^*Apc*^*min/+*^ mice compared with *Apc*^*min/+*^ mice in colonic cancer tissues by IHC staining (Additional file 4: Figure S3C). Collectively, these findings indicated that overexpression of CXCR4 promoted colitis-associated tumorigenesis and progression via recruiting macrophages and MDSCs from the circulatory system to inflamed colonic mucosa.

### The downregulation of miR-133a-3p by activation of CXCL12/CXCR4 plays a crucial role in tumor invasion by targeting RhoA

**Fig. 5:**
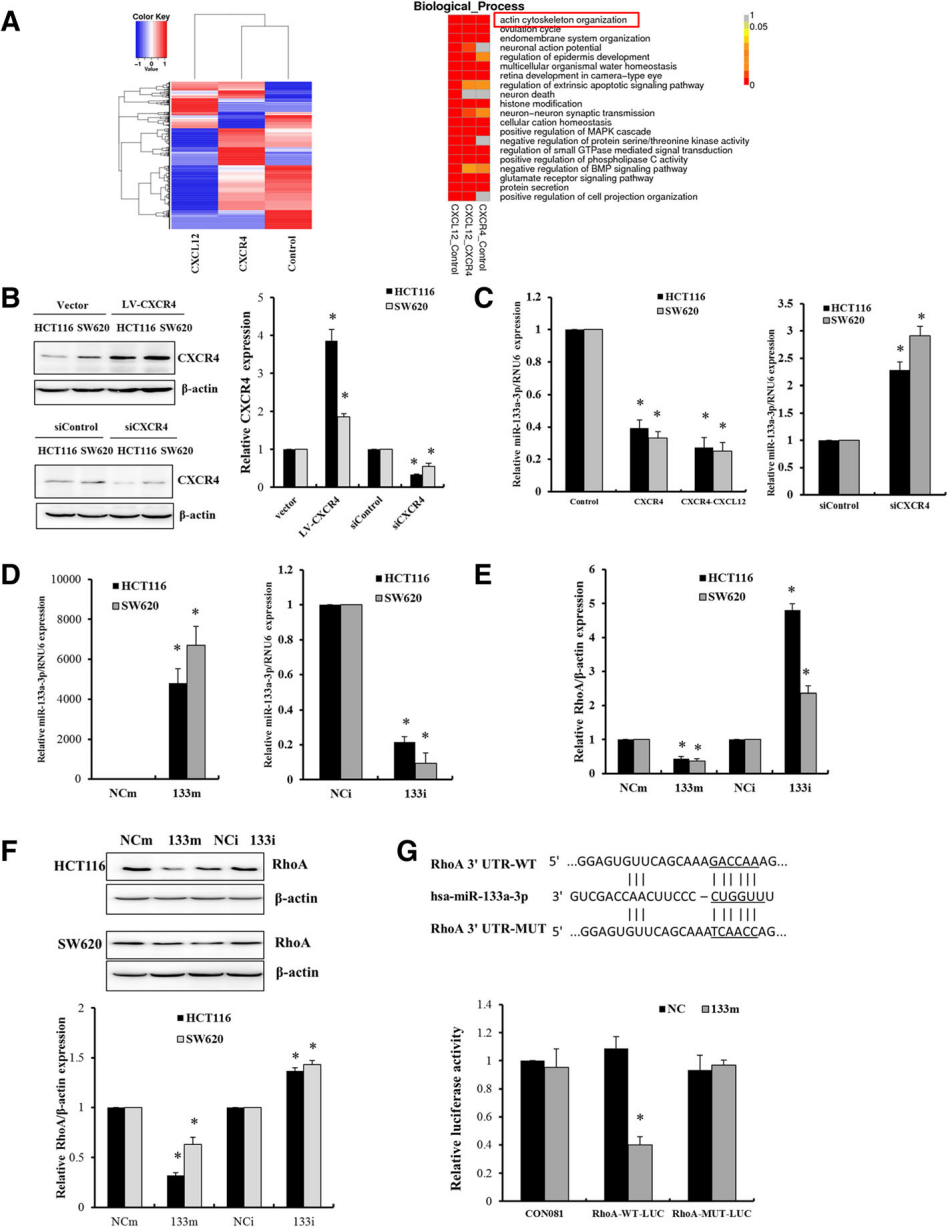
The downregulation of miR-133a-3p by activation of CXCL12/CXCR4 plays a crucial role in the regulation of target gene RhoA. **a** Heatmaps of differentially expressed miRNAs in HCT116 cells overexpressing CXCR4 or induced by 50 ng/μl CXCL12 for 12 h by miRNA-sequencing. GO analysis of the functions of target genes of differentially expressed miRNAs. **b**, **c** HCT116 and SW620 cells infected with lentivirus-CXCR4 were treated with or without 50 ng/μl CXCL12 for 24 h. These cells were also transfected with 200 nM siRNA of CXCR4 for 48 h. Western blot was performed to detect the expression of CXCR4. RT-qPCR was performed to determine the expression of miR-133a-3p. *P < 0.05 vs. control. **d**-**f** HCT116 and SW620 cells were transfected with 100 nM miR-133a-3p mimics (133 m) or inhibitors (133i) for 48 h. The levels of miR-133a-3p and RhoA were determined by RT-qPCR assay and Western blot. **g** HCT116 cells were transfected with luciferase constructs and miR-133a-3p mimics. The comparison of luciferase activity of wild-type (WT) and mutant (MUT) RhoA-3’UTR constructs was performed 36 h after transfection. Data was normalized to renilla activity. *P < 0.05 vs. negative control (NC)

The above results indicated that CXCR4 promotes invasion and progression of inflammatory colorectal cancer. Recent data highlight that miRNAs are closely associated with CRC invasion and metastasis. We identified a variety of differentially expressed miRNAs in colorectal cells upon activation of CXCL12/CXCR4 by miRNA sequencing as shown as heatmaps in Fig. 5a. To our interest, among the eight significantly downregulated miRNAs (Additional file 1: Table S4), miR-133a-3p was the most downregulated miRNA (fold change > 10) in HCT116 cells overexpressing CXCR4 and stimulated by CXC12 compared with vector control cells. GO functional analysis revealed that the target genes of these differentially expressed miRNAs were implicated with actin cytoskeleton reorganization. To further investigate the modulation of miR-133a-3p, colorectal cancer cells HCT116 and SW620 cells were overexpressed of CXCR4 and stimulated by CXCL12, the expression of miR-133a-3p was prominently repressed. In contrast, when CXCR4 was silenced by transfection with CXCR4 siRNA, the expression of miR-133a-3p was robustly upregulated, which suggested that miR-133a-3p was regulated by CXCL12/CXCR4 axis (Fig. 5b, c). To explore the role of miR-133a-3p, Targetscan 7.2 (http://www.targetscan.org) [24] was used to predict the target gene. Importantly, RhoA, a small GTPase protein in the Rho family, was identified to be one of the target genes of miR-133a-3p. Notably, RhoA signaling activates the ROCK family of kinases, promoting the formation of actin stress fibers and generation of the actomyosin contractile force that is required for retraction of the cell rear in mesenchymal-type movement [25]. Therefore, RhoA is a crucial regulator of cancer progression for its contribution to actin cytoskeletal reorganization that drives cell motility and invasion. Colorectal cancer cells were transfected with miR-133a-3p mimics or inhibitors, RhoA expression was examined by RT-QPCR and Western blot. As a result, RhoA was negatively regulated by miR-133a-3p (Fig. 5d-f). Furthermore, luciferase reporter assay was performed to confirm the direct binding of miR-133a-3p with RhoA 3’UTR. However, the binding was abolished by mutation of the binding site of miR-133a-3p on RhoA 3’UTR (Fig. 5g). Collectively, these findings revealed that in response to CXCL12/CXCR4 activation, miR-133a-3p was repressed and implicated with cancer cell motility by upregulating RhoA through direct binding to RhoA 3’UTR.

### CXCL12/CXCR4-induced lncRNA XIST functions as a ceRNA regulating RhoA expression by sponging miR-133a-3p

**Fig. 6:**
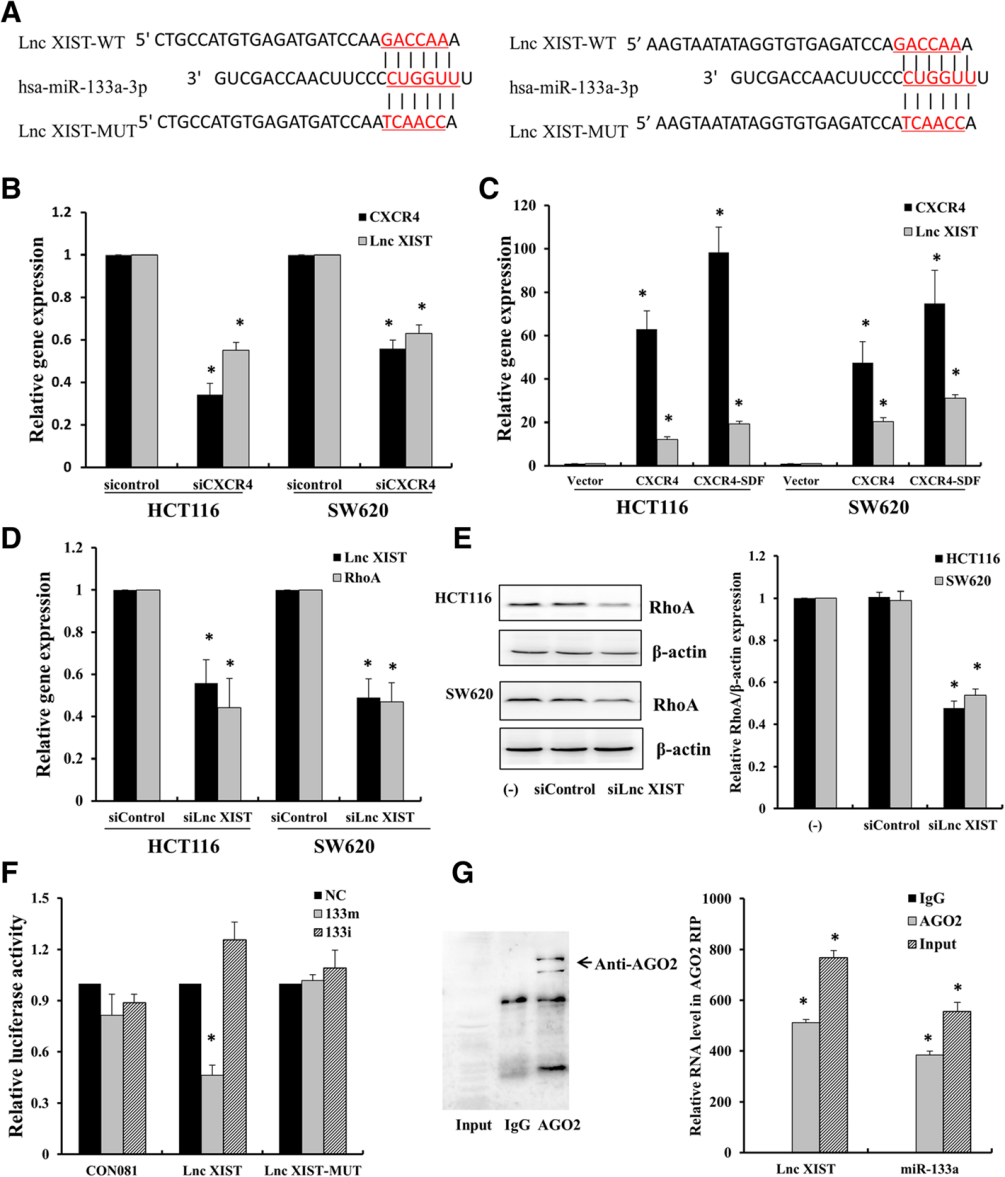
CXCL12/CXCR4-induced lncRNA XIST functions as a ceRNA to regulate Rho A expression by sponging miR-133a-3p. **a** Schematic of binding sites of miR-133a-3p on lncRNA XIST. **b**, **c** HCT116 and SW620 cells were transfected with 200 nM siRNA of CXCR4 for 48 h or overexpressed with CXCR4 with or without 50 ng/μl CXCL12 for 24 h. RT-qPCR was performed to determine the expression of CXCR4 and lncRNA XIST. **d**, **e** HCT116 and SW620 cells were transfected with 200 nM siRNA of lnc RNA XIST for 48 h, the expression of RhoA was determined by RT-qPCR and Western blot. **f** HCT116 cells were transfected with luciferase constructs and miR-133a-3p mimics or inhibitors. The comparison of luciferase activity of wild-type (WT) and mutant (MUT) lncRNA XIST-3’UTR constructs was performed 36 h after transfection. Data was normalized to renilla activity. *P < 0.05 vs. negative control (NC). **g** RNA immunoprecipitation with anti-Ago2 antibody was applied to evaluate endogenous Ago2 binding to RNA. Western blot was used to confirm the expression of Ago2 in anti-Ago2 immunoprecipitate. The level of lncRNA XIST and miR-133a-3p were analyzed by qPCR from Ago2 precipitate. *P < 0.05 vs. IgG control

To investigate the mechanism of suppression of miR-133a-3p upon activation of CXCL12/CXCR4 axis, increasing evidences showed that lncRNAs can function as ceRNAs for miRNAs or naturally occurring miRNA sponges. To identify the key ceRNAs that function as the critical regulators of miR-133a-3p, we used the bioinformatic tools (miRCode, http://www.mircode.org) to search for the potential lncRNAs that can bind to miR-133a-3p. Specifically, lncRNAs XIST were predicted to bind to miR-133a-3p with high conservation. Figure 6a showed the two conservative binding sites of lncRNA XIST with miR-133a-3p. To elucidate whether lncRNA XIST was regulated by activation of CXCL12/CXCR4 axis, CXCR4 was knocked down or overexpressed in HCT116 and SW620 cells induced by CXCL12, consequently, the expression of lncRNA XIST was repressed or elevated accordingly (Fig. 6b, c). Generally, ceRNAs act as molecular sponges for a microRNA through their miRNA binding sites, thereby de-repressing the target genes of miRNA [26]. Thus, we evaluated whether lncRNA XIST can de-repress the expression of RhoA, a target gene of miR-133a-3p, through silencing of lncRNA XIST by siRNA transfection. As a result, knockdown of lncRNA XIST led to a substantial reduction of RhoA both at mRNA and protein levels (Fig. 6d, e). These results indicated that the expression of lncRNA XIST was positively associated with the expression of RhoA.

To further confirm the direct binding between lncRNA XIST and miR-133a-3p, we constructed lncRNA XIST luciferase reporter plasmids with miR-133a-3p binding site (lncRNA XIST-WT) and its mutant type (lncRNA XIST-MUT). Cotransfection of lncRNA XIST-WT with miR-133a-3p significantly reduced the luciferase activity compared with miR-NC, whereas co-transfection of lncRNA XIST-MUT with miR-133a-3p did not show significant change in luciferase activity (Fig. 6f). miRNA is known to be present in the cytoplasm in the form of miRNA ribonucleoprotein complex (miRNP) containing argonaute-2 (AGO2), the core component of RISC [27]. To determine if lncRNA XIST is binding with miRNP, RIP experiment was conducted by using anti-AGO2 antibody. LncRNA XIST and miR-133a-3p were significantly enriched in AGO2-containing immunoprecipitates compared with control immunoglobulin G (IgG) immunoprecipitates. Successful immunoprecipitation of AGO2 was confirmed by Western blot (Fig. 6g).

### CXCR4 enhanced CRC invasion by regulating cytoskeletal reorganization through activation of RhoA

**Fig. 7:**
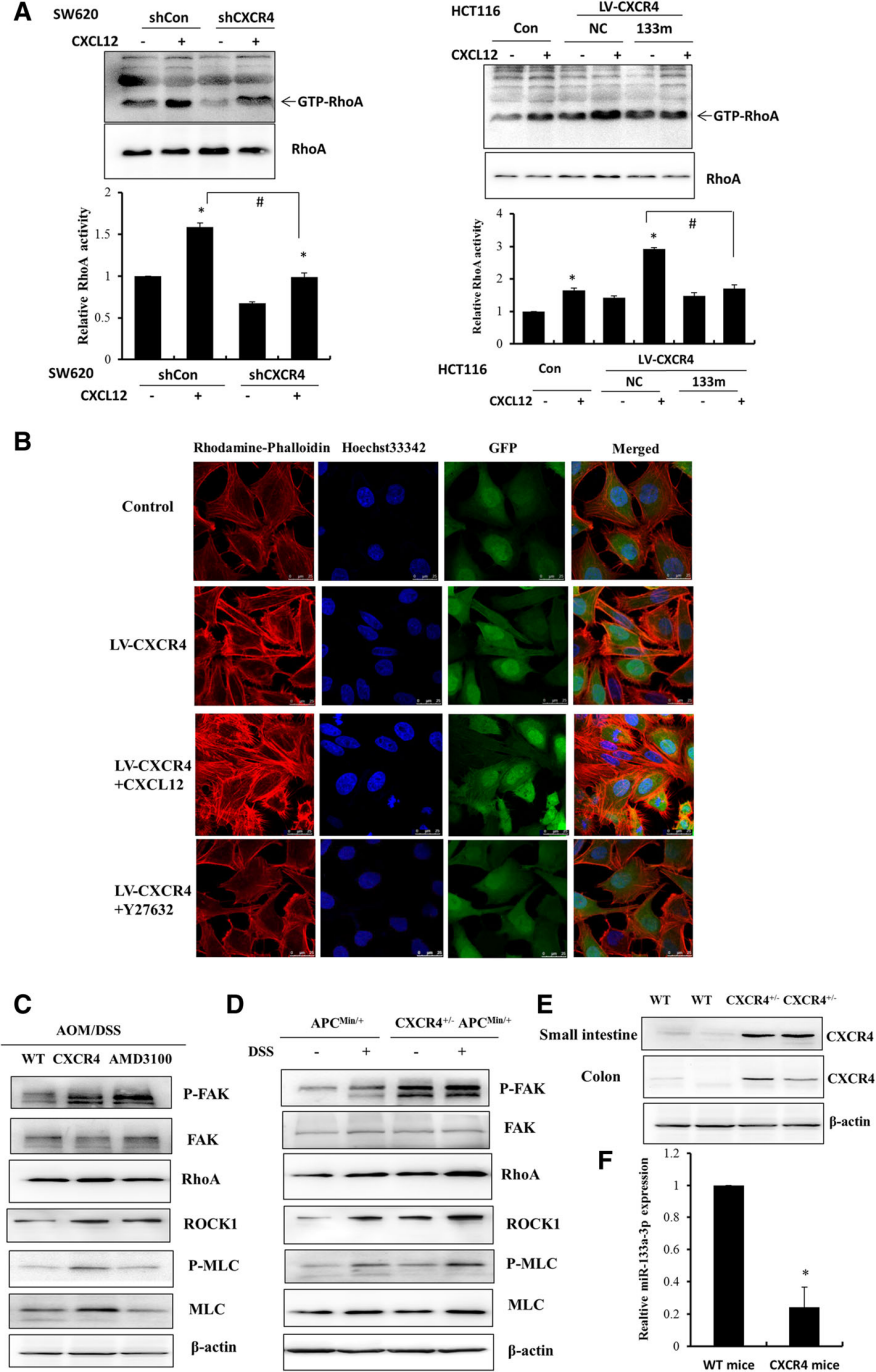
CXCR4 promotes the invasion of CRC by regulation of cytoskeletal assembly through activation of RhoA. **a** CXCR4 was knocked down by infection with CXCR4 shRNA in SW620 cells, CXCR4 was overexpressed by infection with LV-CXCR4 in HCT116 cells with or without the treatment of 50 ng/μl CXCL12 for 12 h. RhoA activity (GTP-RhoA) was determined by GST pull down assay. HCT116 cells overexpressing CXCR4 were transfected with 100 nM miR-133a-3p, RhoA activity was examined and statistical analyses were performed compared with control. *P < 0.05 vs. control, #*P* < 0.05 vs.CXCL12-treated group as indicated. **b** HCT116 cells overexpressing CXCR4 were treated with 50 ng/μl CXCL12 or ROCK inhibitor Y27632 for 24 h, Rhodamine-labeled Phalloidin was used to indicate actin polymerization. DAPI showed nuclear staining. GFP was used to indicate the transfection with LV-CXCR4-GFP or LV-Control-GFP. The images were observed under confocal microscope. **c**, **d** Western blot was performed to detect the expression levels of p-FAK(Y925)/FAK, RhoA, ROCK1, p-MLC/MLC normalized to β-actin in AOM/DSS treated WT, *CXCR4*^*+/−*^ mice with or without AMD3100 as well as *Apc*^*Min/+*^ and *CXCR4*^*+/−*^ *Apc*^*Min/+*^ compound mice treated with or without DSS. **e** Western blot was performed to detect the expression of CXCR4 in small intestine and colon tissues of WT and *CXCR4*^*+/−*^ mice. **f** The expression of miR-133a-3p was examined in colon tissue of WT and *CXCR4*^*+/−*^ mice by RT-qPCR

The above results demonstrated that CXCL12/CXCR4-induced lncRNA XIST could function as a ceRNA interfering with the regulation of RhoA by miR-133a-3p. Since miR-133a-3p was significantly reduced upon stimulation of CXCL12/CXCR4 axis, it is likely that the activation of RhoA was prominently enhanced to modulate actin cytoskeletal assembly and cell motility. To confirm this result, in vitro RhoA kinase assay was performed in HCT116 and SW620 cells by knockdown and overexpression of CXCR4 in response to CXCL12. As shown in Fig. 7a, knockdown of CXCR4 in SW620 cells robustly suppressed the activation of RhoA, although with a moderate increase upon the stimulation of CXCL12. In contrast, overexpression of CXCR4 in HCT116 cells strongly induced the activation of RhoA, which can be potentiated by CXCL12. Importantly, transfection of miR-133a-3p mimics attenuated RhoA activity in CXCR4- overexpressing HCT116 cells, indicating that activation of CXCL12/CXCR4 axis enhanced RhoA activity through downregulation of miR-133a-3p. Next, we investigated whether CXCR4 promoted the invasion of CRC by regulation of actin cytoskeleton organization through activation of RhoA/ROCK signaling. RhoA signals to the ROCK kinase, promoting the formation of actin stress fibers and generation of the actomyosin contractile force required for cell movement [28]. As shown in Fig. 7b, immunofluorescence intensity of F-actin labeled by rhodamine-labeled phalloidin was robustly enhanced in CXCR4 overexpressing HCT116 cells, and CXCL12 further enhanced the actin polymerization and exhibited more protrusions in the invasive front of cells. Importantly, this phenomenon can be greatly abrogated by ROCK inhibitor Y-27632, suggesting that RhoA/ROCK signaling was involved in CXCL12/CXCR4-induced actin cytoskeletal reorganization and assembly.

ROCK inactivates myosin phosphatase by direct phosphorylation and activation of myosin light chain (MLC). As a consequence, RhoA/ROCK/p-MLC signaling enhances actomyosin contractility driving cell motility. To prove that CXCR4 plays a crucial role in colorectal cancer invasion through activation of RhoA/ROCK signaling in vivo, Western blot was performed to examine the expression of RhoA, p-FAK/FAK, ROCK1 as well as p-MLC/MLC in colonic tissues of AOM/DSS-induced *CXCR4*^*+/−*^ mice and *Apc*^*min/+*^ mice. The expression of RhoA, ROCK1 were markedly increased and p-FAK, p-MLC were significantly enhanced in *CXCR4*^*+/−*^ mice, which can be attenuated by AMD3100. Consistently, CXCR4 enhanced the expression of RhoA, ROCK1 and phosphorylation of FAK, MLC in *Apc*^*min/+*^ mice, particularly in response to DSS treatment (Fig. 7c, d). Importantly, the expression of miR-133a-3p was substantially reduced in the colonic mucosa of *CXCR4*^*+/−*^ mice compared with that of WT mice (Fig. 7e, f). Further functional experiments of miR-133a demonstrated that overexpression of miR-133a suppressed EMT and cell invasion in CRC (Additional file 5: Figure S4). These results indicated that CXCR4 plays a critical role in the activation of RhoA-ROCK/p-MLC signaling that was responsible for actin polymerization and CRC cell invasion through downregulation of miR-133a-3p.

**Fig. 8:**
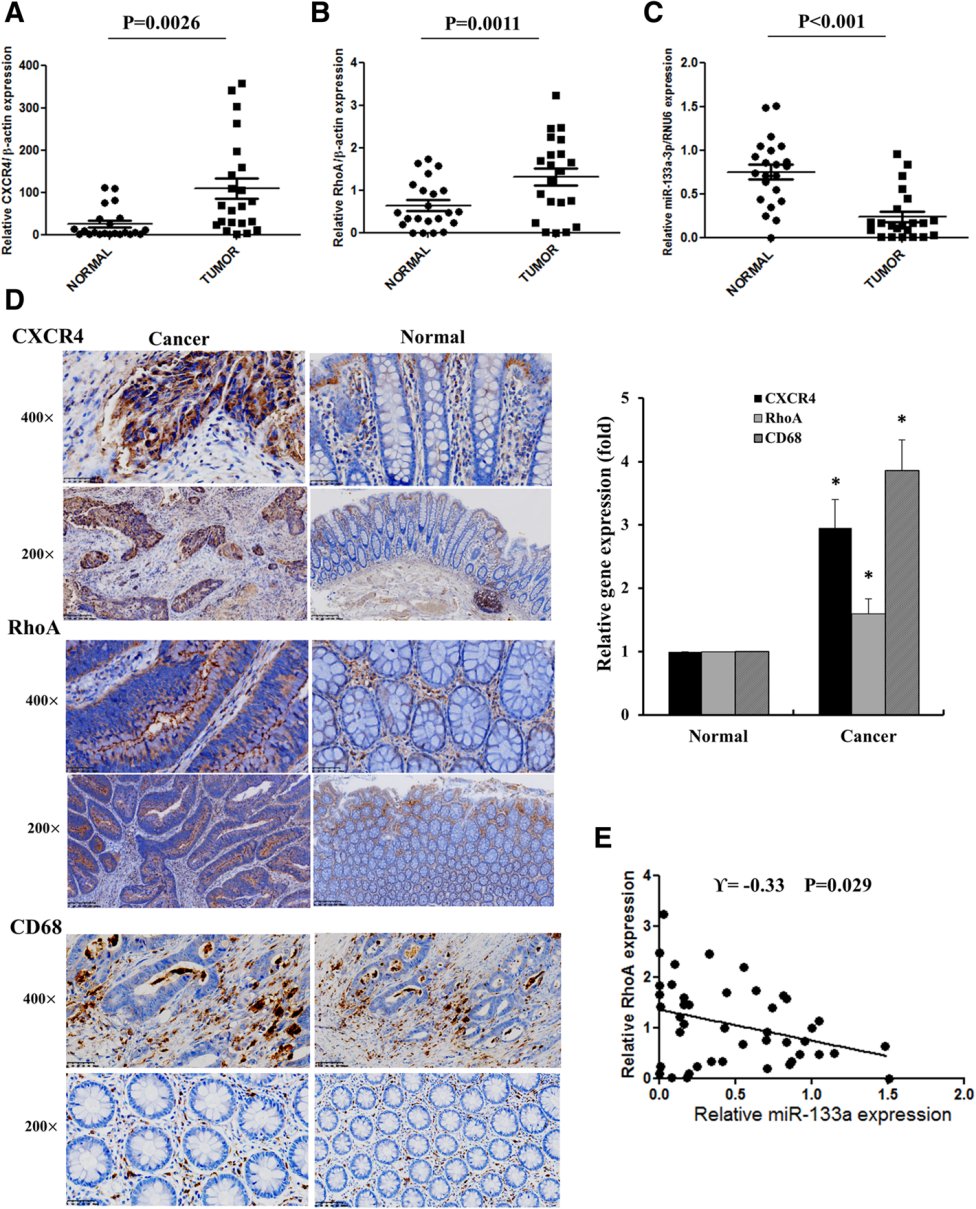
miR-133a-3p was negatively associated with the expression of RhoA in human CRC specimens. **a**-**c** Statistical analysis of the mRNA level of CXCR4, RhoA and miR-133a-3p in 22 pairs of human colon tissues and adjacent normal colon tissue. **d** The expression and location of CXCR4, RhoA and CD68 were analyzed by IHC assay in paired human CRC tissues and statistical analysis was performed. *P < 0.05 vs. normal colonic tissues. **e** The negative correlation between RhoA and miR-133a-3p expression was detected by Spearman’s correlation analysis in human CRC tissues

To extend current knowledge to colorectal cancer patients, we collected 22 pairs of human CRC specimens and adjacent normal colon tissues for immunohistochemistry and RT-QPCR analysis. Both mRNA and protein levels of CXCR4 and RhoA were substantially elevated in CRC tissues compared with normal colon tissues. Moreover, CXCR4 was remarkably expressed in the cytoplasm and membrane of colon cancer cells and RhoA was predominantly expressed in the cytoplasm of colorectal cancer tissues. CD68, the surface marker of macrophages, was highly expressed in human CRC tissues compared with normal colon tissues, indicating the infiltration of more macrophages in CRC tissues. As expected, miR-133a-3p was robustly downregulated in colorectal cancer tissues compared with normal colon tissues (Fig. 8a-d). There was a strong inverse correlation between miR-133a-3p and RhoA expression (Fig. 8e). Taken together, these data suggest that in clinical colorectal cancer patients high CXCR4 expression contribute to CRC progression and invasion through recruiting macrophages and upregulation of RhoA by repressing miR-133a-3p.

## Discussion

**Fig. 9:**
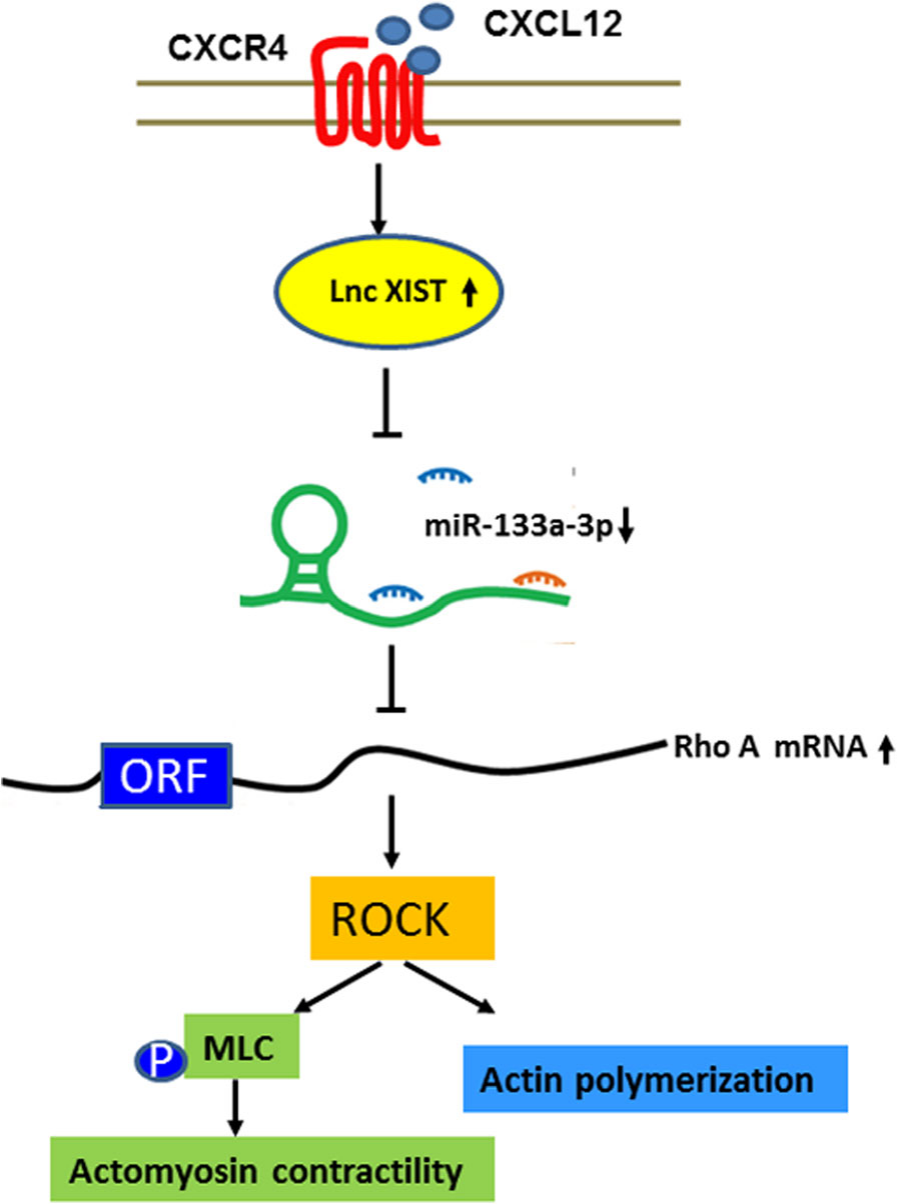
Schematic of proposed molecular model of non-coding RNAs involved in CXCL12/CXCR4-induced CRC invasion and progression. Activation of CXCL12/CXCR4 axis upregulates LncRNA XIST. LncRNA XIST functions as a ceRNA sponging miR-133a-3p, thereby upregulating RhoA, which promoted actin polymerization and actomyosin contractility through the ROCK/p-MLC pathway

Although it has been proposed that CXCR4 plays a crucial role in migration, invasion and liver metastasis of CRC [2], the mechanism of activation of CXCL12/CXCR4 on inflammation-driven CRC progression remains largely unknown. Some in vitro studies showed that CXCL12/CXCR4 promoted the migration and liver metastasis of CRC by upregulating αvβ6 through ERK/Ets-1 [29] and Wnt/β-catenin signaling pathway [4]. However, the association of CXCR4 with invasion and metastasis of CRC was almost exclusively from clinical pathological data and CRC cell lines. To our knowledge, it is the first time that we utilized villin-CXCR4 transgenic mice model to prove that overexpression of CXCR4 indeed could increase both AOM/DSS-induced CAC and *Apc* mutation-driven tumorigenesis and progression. Although we did not observe liver metastasis in these animal models, orthotopic implantation of HCT116 cells overexpressing CXCR4 into the cecum wall of nude mice produced higher incidence of spontaneous hepatic metastases compared with control cells (data not shown). Herein, using both villin-CXCR4 transgenic mice and human CRC specimens, we demonstrated that CXCR4 promotes the progression of CAC by enhancing EMT and recruiting MDSCs and macrophages. Furthermore, we illustrated that miR-133a-3p was downregulated upon the activation of CXCL12/CXCR4 axis. lncRNA XIST functions as a ceRNA sponging miR-133a-3p, thereby de-repressing target gene of RhoA (Fig. 9). These results uncover the important role of lncRNA XIST/ miR-133a-3p/ RhoA signaling on progression of CAC induced by CXCL12/CXCR4 activation, which provides potential therapeutic targets for preventing CRC invasion and liver metastasis.

Chronic inflammation and inflammatory mediators promote tumor initiation, invasion and metastasis. MDSC and macrophages are important inflammatory drivers of immune suppression and established cross-talk impacting anti-tumor immunity in most tumors [30]. MDSCs potentially inhibit T cell function and activation that correlates well with clinical cancer stage and metastatic tumor burden in CRC patients [31]. Tumour associated macrophages (TAMs) have been reported to play a key role in the progression of CAC by secreting vascular endothelial growth factor (VEGF) and are linked with poor prognosis [32]. Using villin-CXCR4 transgenic mice, we provide strong evidence that overexpression of CXCR4 enhanced the recruitment of MDSCs especially G-MDSCs and macrophages in the colonic epithelial mucosa to trigger the progression of CAC. Consistently, it was reported that CXCL12/CXCR4 axis triggered PGE2-induced accumulation of MDSCs in ovarian cancer microenvironment [33]. Multiple myeloma cells recruit tumor-supportive macrophages through the CXCL12/CXCR4 axis and promote their polarization toward M2 phenotype [34]. Our results unveiled a mechanism of the recruitment of MDSCs and TAM in colonic epithelial mucosa which contributed to the progression of CAC.

To explore the potential miRNAs and related lncRNAs that play an important role in CXCL12/CXCR4-induced invasion and metastasis of CRC, we screened the differentially expressed miRNAs upon the activation of CXCL12/CXCR4 axis in CRC cell models. Notably, miR-133a-3p was found to be significantly reduced in HCT116 cells overexpressing CXCR4. Increasing evidences demonstrated that CXCL12/CXCR4 promoted EMT and tumor invasion in CRC [4, 35], miR-133a-3p was markedly downregulated in a variety of cancers and associated with tumorigenesis and metastasis [36, 37]. We firstly demonstrated that RhoA was the direct target gene of miR-133a-3p. More importantly, the negative correlation of RhoA and miR-133a-3p was observed in human CRC specimens. Therefore, it is likely that the downregulated miR-133a-3p upon the activation of CXCL12/CXCR4 axis promoted the invasion and metastasis of CRC by regulation of target gene RhoA.

In addition, we investigated the mechanism of downregulation of miR-133a-3p by the activation of CXCL12/CXCR4 axis. LncRNAs were recently reported to function as ceRNAs that could sponge miRNAs. We predict that lncRNA XIST is the potential ceRNA of miR-133a-3p and further confirmed that lncRNA XIST regulated the expression of RhoA through sponging miR-133a-3p determined by the assays of luciferase activity and RIP. Lnc RNA XIST has been reported to exert the oncogenic functions in many cancers by acting as a molecular sponge of miR-34, miR-101 and miR-181a [15, 38, 39]. We unveiled a novel mechanism of downregulation and repression of miR-133a-3p by CXCL12/CXCR4-induced lnc RNA XIST.

Tumor invasion is a complex and multistep program involved in the interplay of tumor cells and the microenvironment, and in turn tumor cells acquire the capacity of migration and invasion. RhoA signaling is engaged in at least two distinct types of motility in three-dimensional matrix, amoeboid motility and mesenchymal motility [40]. RhoA regulates cell motility through cytoskeletal reorganization, and has been reported to be dysregulated in CRC [41, 42]. GTP binding and hydrolysis switches RhoA between a GTP-bound active and a GDP-bound inactive state. ROCK is a principal mediator of cytoskeletal tension downstream of RhoA. ROCK enhances actomyosin contractility by direct phosphorylation and activation of myosin light chain (MLC) [43]. Compared with WT mice, the upregulation of RhoA, ROCK1 and p-MLC in *CXCR4*^*+/−*^ transgenic mice induced by AOM/DSS could be impaired by AMD3100. These results demonstrated that RhoA/ROCK/p-MLC signaling played an important role in the cytoskeletal assembly and migration of CAC in *CXCR4*^*+/−*^ transgenic mice. Accumulating evidences have indicated a prominent role of RhoA/ROCK signaling in the progression and metastasis of multiple tumors [44, 45]. To confirm that activation of CXCL12/CXCR4 is truly involved in the activation of RhoA, active form of GTP-RhoA was found to be regulated by the activation of CXCL12/CXCR4 axis in colorectal cancer cell model. Importantly, CXCL12/CXCR4-induced actin polymerization and reorganization was driven by the active RhoA, which can be blocked by ROCK inhibitor Y27632.

Taken together, our results showed that overexpressing CXCR4 in the intestine epithelial mucosa accelerates the tumorigenic process in inflammation-driven tumorigenesis, resulting in massive infiltration of inflammatory MDSCs and macrophages and increased tumor size and multiplicity. To further explore the underlying mechanism, miRNA sequencing results showed that miR-133a-3p was significantly downregulated in response to CXCL12/CXCR4 axis. We then used in vitro models to demonstrate that in CRC cells, miR-133a-3p directly targeted the regulation of RhoA that triggered tumor motility/invasion. More importantly, lncRNA XIST function as a ceRNA that sponged miR-133a-3p and liberated the repression of RhoA by miR-133a-3p. The regulation of lncRNA XIST/ miR-133a-3p/RhoA/ROCK/P-MLC signaling pathway was further supported in both human CRC specimens and CXCR4^+/−^ transgenic mice models.

## Conclusions

Our results highlight the important role of CXCR4 in promoting inflammation-driven colorectal cancer progression through activation of RhoA/ROCK signaling by lncRNA XIST mediated sponging of miR-133a. These results shed a new insight into the role of miR-133a-3p in CXCL12/CXCR4-induced invasion and metastasis of CRC, which provides potential therapeutic targets for hindering the metastatic behavior.

## Additional files

Additional file 1: **Table S1.** Primers for genotyping of CXCR4 transgenic mice and Apc^Min/+^ mice. **Table S2.** Antibodies for identification of the immunocytes. **Table S3.** Clinical pathological analysis of human CRC specimens. **Table S4.** The downregulated miRNAs in HCT116 cells upon activation of CXCL12/CXCR4 by miRNA sequencing analysis. (DOCX 20 kb)

Additional file 2: **Figure S1.** Representative genotyping results of CXCR4 transgenic mice and *Apc*^*min/+*^ mice were performed by PCR assay. The PCR products (CXCR4 and β-actin as well as wild type and mutant Apc) were run by agarose gel electrophoresis. (A) The mice were all CXCR4 transgenic mice except No. 3, 19, 23, 25, 29, 32, 35 that were WT mice. (B) The *Apc*^*min/+*^ mice were No. 1, 3, 4, 6, 7. (TIF 663 kb)

Additional file 3: **Figure S2.** CXCR4 increased tumorigenesis in *Apc*^*min/+*^ mice in a time-dependent manner. *Apc*^*min/+*^ and *CXCR4*^*+/−*^*Apc*^*min/+*^ compound mutant mice were treated with or without DSS. At the ages of 22 and 26 weeks, the mice were sacrificed and representative images of intestine polyps were shown. (TIF 3277 kb)

Additional file 4: **Figure S3.** The percentages of gated CD4^+^, CD8^+^ T-cells, CD11b^+^F4/80^+^ macrophages, CD11b^+^Ly6C^+^, CD11b^+^Ly6G^+^ MDSCs immune cells in the blood (A) and colonic tissues (B) of CXCR4^+/−^*Apc*^*min/+*^ and *Apc*^*min/+*^ mice treated with or without DSS were subjected to flow cytometry analysis. (C) The staining of CD8^+^ T cells were performed by IHC assay and statistical analysis were performed (*n* = 3). *P < 0.05 vs. *Apc*^*min/+*^ mice. (TIF 2429 kb)

Additional file 5: **Figure S4.** MiR-133a inhibited invasion in CRC cells. (A) SW620 and HCT116 cells were transfected with 100 nM miR-133a-3p mimics (133 m) or inhibitors (133i) for 48 h. The levels of vimentin and E-cadherin were determined by Western blot. (B) SW620 and HCT116 cells were transfected with 100 nM miR-133a-3p mimics (133 m) for 24 h, invasion of cells was examined by transwell assay. (TIF 726 kb)

## Abbreviations

AOM/DSS: Azoxymethane/dextran sulfate sodium
ceRNAs: Competitive endogenous RNAs
CRC: Colorectal cancer
CXCR4: Chemokine (C-X-C motif) receptor 4
EMT: Epithelial-mesenchymal transition
HE: Hematoxylin & eosin
IEC: Intestinal epithelial cells
lncRNAs: Long non-coding RNAs
MDSCs: Myeloid-derived suppressor cells
miRNA: MicroRNA
PRC2: Polycomb repressive complex 2
RISC: RNA-induced silencing complex
SDF-1: Stromal cell-derived factor 1
XIST: LncRNA X-inactive-specific transcript
