# Supplementary material for: CXCL12/CXCR4 promotes inflammation-driven colorectal cancer progression through activation of RhoA signaling by sponging miR-133a-3p
Source: J Exp Clin Cancer Res. 2019 Jan 24;38:32. doi: 10.1186/s13046-018-1014-x (PMC6346552; doi:10.1186/s13046-018-1014-x)
Supplement: Supplementary file 1 — Table S1. Primers for genotyping of CXCR4 transgenic mice and ApcMin/+ mice. Table S2. Antibodies for identification of the immunocytes. Table S3. Clinical pathological analysis of human CRC specimens. Table S4. The downregulated miRNAs in HCT116 cells upon activation of CXCL12/CXCR4 by miRNA sequencing analysis. (DOCX 20 kb) [file 13046_2018_1014_MOESM1_ESM.docx]

Additional file 1

**Table S1. Primers for genotyping of CXCR4 transgenic mice and Apc^Min/+^ mice**

|  | Forward primer (5′-3′) | Reverse primer (5′-3′) |
| --- | --- | --- |
| CXCR4^+/-^ mice | CTTCTCCTCTAGGCTCGTCCAC | GATCACCAATCCATTGCCGACT |
| WT mice | ACTCCAAGGCCACTTATCACC | ATTGTTACCAACTGGGACGACA |
| Apc^Min/+^ mice (mut allele) | TTCCACTTTGGCATAAGGC | TTCTGAGAAAGACAGAAGTTA |
| Apc^Min/+^ mice (wt allele) | GCCATCCCTTCACGTTAG | TTCCACTTTGGCATAAGGC |

**Table S2. Antibodies for identification of the immunocytes**

| **Antibodies** | **Usage** | **Immune cell types** | **Companies** |
| --- | --- | --- | --- |
| CD11b^+^Ly6C^+^ (101206, 128008) | 0.25 µg/10^6^ cells, 0.25 µg/10^6^ cells | M-MDSCs | Biolegend |
| CD11b^+^Ly6G^+^ (101206, 127627) | 0.25 µg/10^6^ cells, 0.5 µg/10^6^ cells | G-MDSC | Biolegend |
| CD3^+^/CD4^+^ (100234, 100424) | 0.5 µg/10^6^ cells, 0.25 µg/10^6^ cells | CD4^+^ T lymphocytes | Biolegend |
| CD3^+^/CD8^+^ (100234, 100730) | 0.5 µg/10^6^ cells, 0.25 µg/10^6^ cells | CD8^+^ T lymphocytes | Biolegend |
| CD11b^+^F4/80^+^(101206, 123128) | 0.25 µg/10^6^ cells, 1.0 µg/10^6^ cells | macrophages | Biolegend |

**Table S3. Clinical pathological analysis of human CRC specimens**

| Variables | Classification | Case numbers (n = 22) |
| --- | --- | --- |
| Sex | Male | 12 |
|  | female | 10 |
| Age | <=70 | 11 |
|  | >70 | 11 |
| Tumor location | Colon | 19 |
|  | rectum | 3 |
| Histology | tubular adenocarcinoma | 16 |
|  | [mucinous adenocarcinoma](http://www.baidu.com/link?url=AkN5NSxNhQUVLL6kGF7S8zR7nSHEdjOJwLNURWqD6ZeX5nPUCL4AmhsKRE031oBeTnz1zhT2gErBm30DTRtmnxLcn4Op36KuoLb-4MfSowIMVZmQDnwhMcLcP1JkoOwO) | 2 |
|  | Mixed adenocarcinoma | 4 |
| Differentiation | low | 5 |
|  | Moderate and high | 17 |
| TNM stages | T2 | 1 |
|  | T3 | 15 |
|  | T4 | 6 |
|  | N0 | 12 |
|  | N1 | 8 |
|  | N2 | 2 |
|  | M0 | 16 |
|  | M1 | 6 |
| Liver metastasis | negative | 18 |
|  | positive | 4 |
| Microsatellite instability (MSI) | negative | 18 |
|  | positive | 4 |

**Table S4. The downregulated miRNAs in HCT116 cells upon activation of CXCL12/CXCR4 by miRNA sequencing analysis**

| miRNA | CXCR4 | Control | Fold | *P* value | Significant | |
| --- | --- | --- | --- | --- | --- | --- |
| hsa-miR-133a-3p | 1.407838 | 17.33086 | 0.081233 | 1.15E-19 | yes |  |
| hsa-miR-3155b | 0.10056 | 3.563355 | 0.028221 | 2.04E-05 | yes |  |
| hsa-miR-6511a-3p | 3.620154 | 8.422475 | 0.429821 | 0.00355274 | yes |  |
| hsa-miR-195-3p | 1.206718 | 4.049267 | 0.298009 | 0.00922137 | yes |  |
| hsa-miR-3138 | 1.608957 | 4.69715 | 0.342539 | 0.01025637 | yes |  |
| hsa-miR-451a | 0.10056 | 1.295765 | 0.077607 | 0.03197384 | yes |  |
| hsa-miR-584-3p | 0.402239 | 1.943648 | 0.206951 | 0.0405807 | yes |  |
| hsa-miR-4512 | 0.603359 | 2.267589 | 0.266079 | 0.04926713 | yes |  |
