# Supplementary figures and images for: CXCL12/CXCR4 promotes inflammation-driven colorectal cancer progression through activation of RhoA signaling by sponging miR-133a-3p
Source: J Exp Clin Cancer Res. 2019 Jan 24;38:32. doi: 10.1186/s13046-018-1014-x (PMC6346552; doi:10.1186/s13046-018-1014-x)

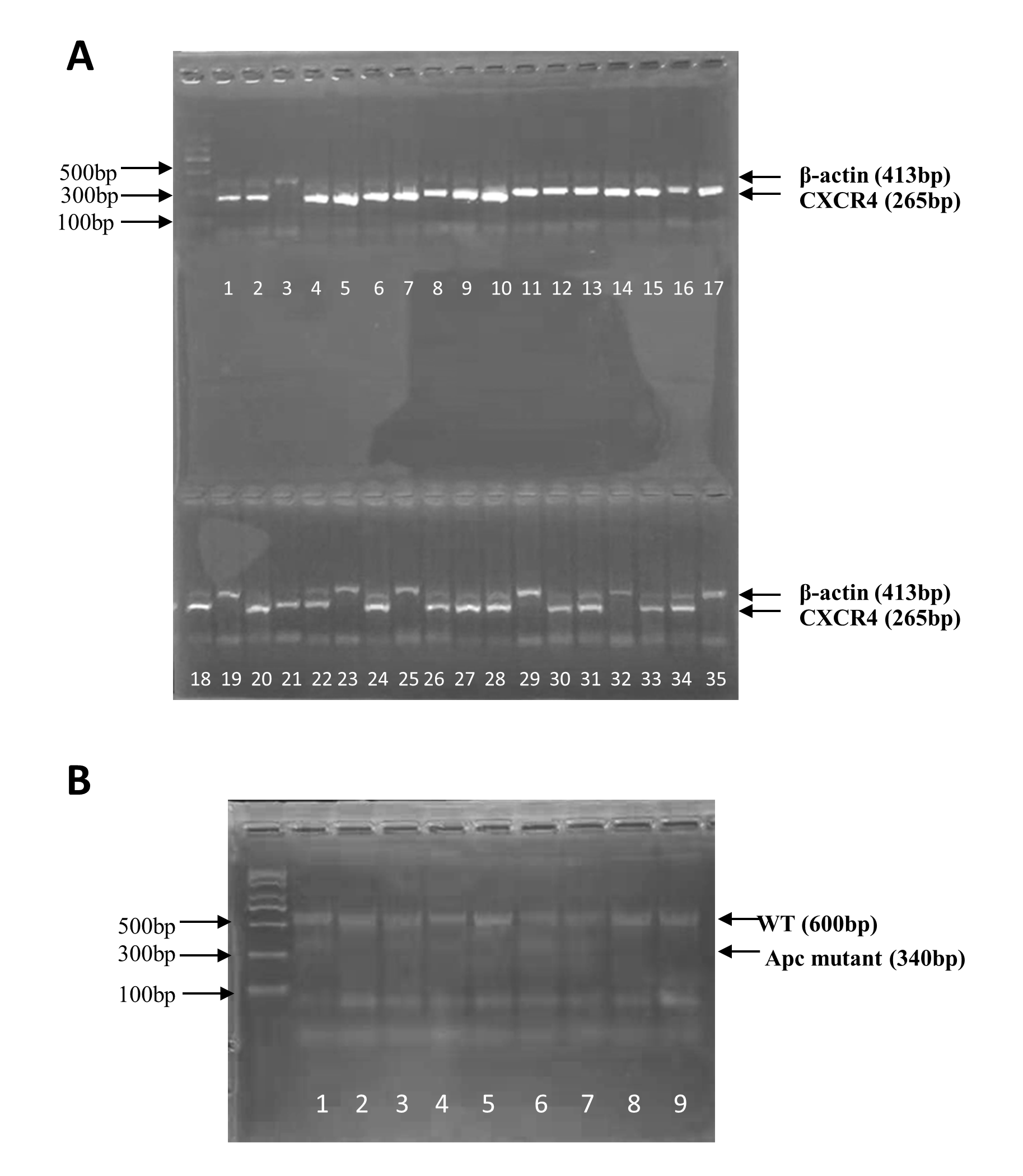

Supplement: Supplementary file 2 — Figure S1. Representative genotyping results of CXCR4 transgenic mice and Apcmin/+ mice were performed by PCR assay. The PCR products (CXCR4 and β-actin as well as wild type and mutant Apc) were run by agarose gel electrophoresis. (A) The mice were all CXCR4 transgenic mice except No. 3, 19, 23, 25, 29, 32, 35 that were WT mice. (B) The Apcmin/+ mice were No. 1, 3, 4, 6, 7. (TIF 663 kb) [file 13046_2018_1014_MOESM2_ESM.tif]

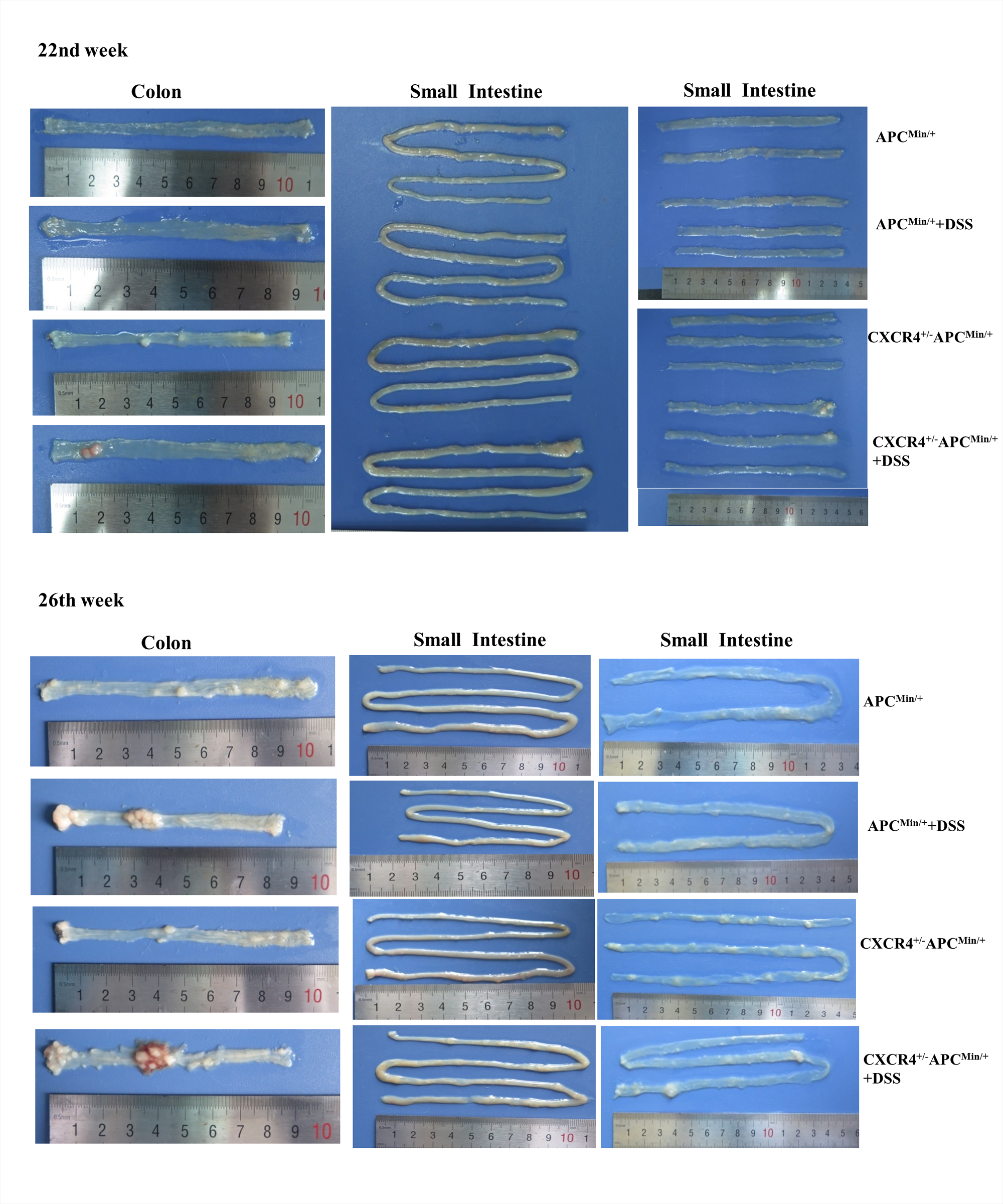

Supplement: Supplementary file 3 — Figure S2. CXCR4 increased tumorigenesis in Apcmin/+ mice in a time-dependent manner. Apcmin/+ and CXCR4+/−Apcmin/+ compound mutant mice were treated with or without DSS. At the ages of 22 and 26 weeks, the mice were sacrificed and representative images of intestine polyps were shown. (TIF 3277 kb) [file 13046_2018_1014_MOESM3_ESM.tif]

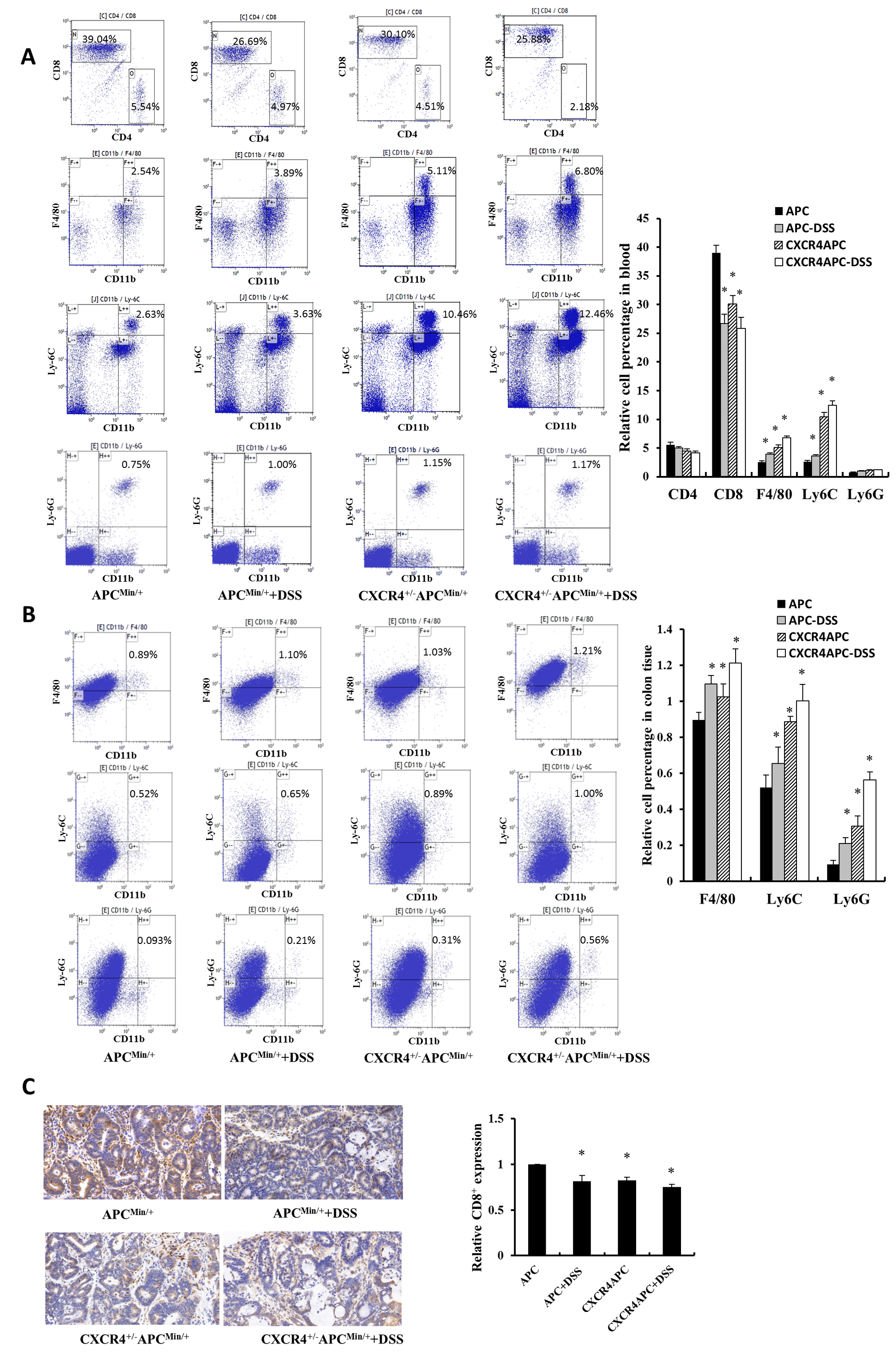

Supplement: Supplementary file 4 — Figure S3. The percentages of gated CD4+, CD8+ T-cells, CD11b+F4/80+ macrophages, CD11b+Ly6C+, CD11b+Ly6G+ MDSCs immune cells in the blood (A) and colonic tissues (B) of CXCR4+/−Apcmin/+ and Apcmin/+ mice treated with or without DSS were subjected to flow cytometry analysis. (C) The staining of CD8+ T cells were performed by IHC assay and statistical analysis were performed (n = 3). *P < 0.05 vs. Apcmin/+ mice. (TIF 2429 kb) [file 13046_2018_1014_MOESM4_ESM.tif]

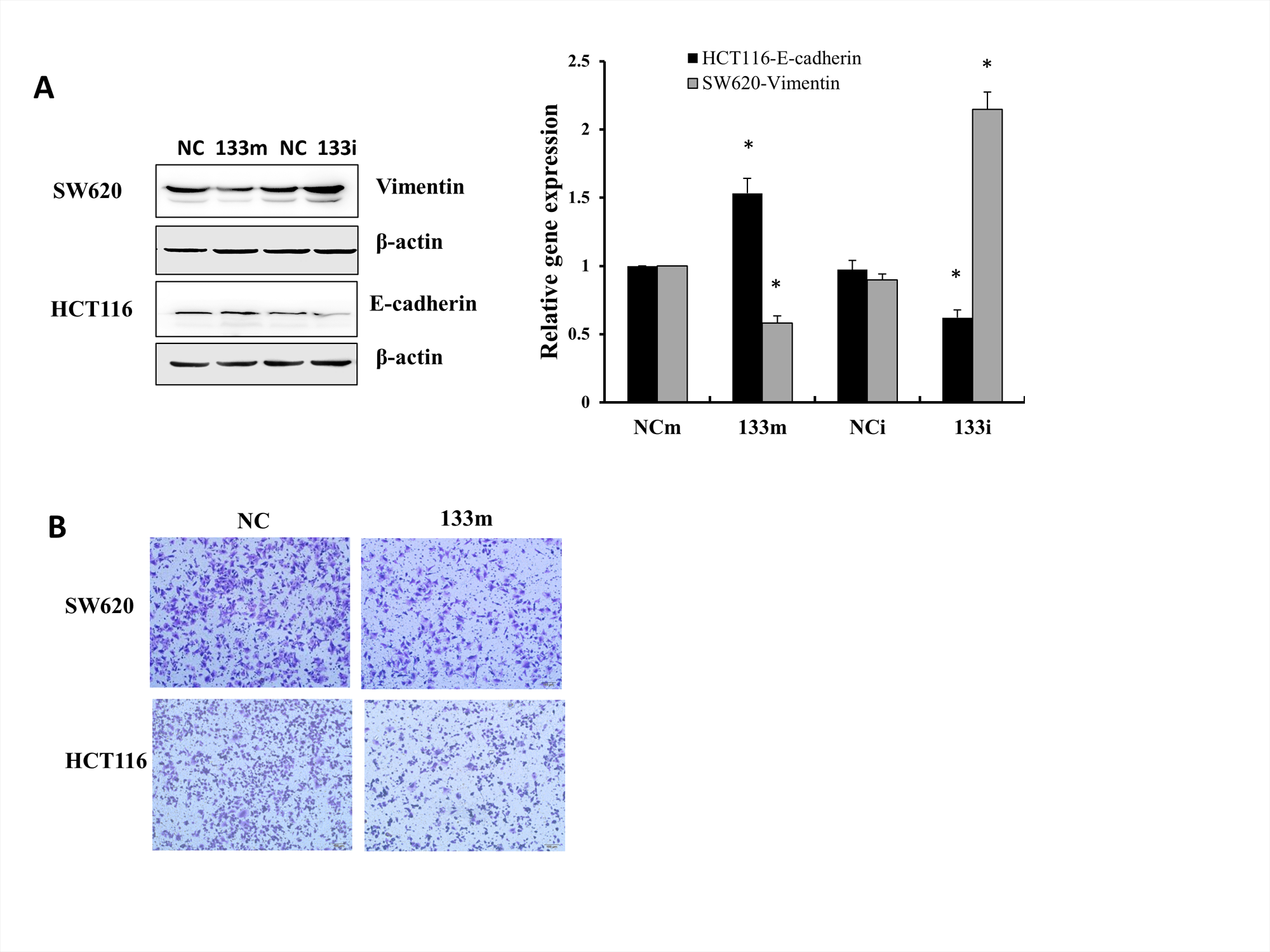

Supplement: Supplementary file 5 — Figure S4. MiR-133a inhibited invasion in CRC cells. (A) SW620 and HCT116 cells were transfected with 100 nM miR-133a-3p mimics (133 m) or inhibitors (133i) for 48 h. The levels of vimentin and E-cadherin were determined by Western blot. (B) SW620 and HCT116 cells were transfected with 100 nM miR-133a-3p mimics (133 m) for 24 h, invasion of cells was examined by transwell assay. (TIF 726 kb) [file 13046_2018_1014_MOESM5_ESM.tif]
